# Supplementary material for: A study of association between maternal tetanus toxoid immunization and neonatal mortality in the context of Bangladesh
Source: PLoS One. 2025 Jan 17;20(1):e0316939. doi: 10.1371/journal.pone.0316939 (PMC11741588; doi:10.1371/journal.pone.0316939)
Supplement: S1 Table — (DOCX) [file pone.0316939.s001.docx]

S1 Table. Analysis results for examining neonatal mortality against Tetanus doses taken by mothers.

| **Characteristics** | **Crude IRR (95% CI)** | **p-value** | **Adjusted IRR (95% CI)** | **p-value** |
| --- | --- | --- | --- | --- |
| **TT doses taken** |  |  |  |  |
| None | 1 |  | 1 |  |
| 1 injection | 0.84(0.64,1.09) | 0.197 | 0.77(0.54,1.11) | 0.160 |
| 2 injections or more (adequate doses) | 0.87(0.57,1.32) | 0.498 | 0.54(0.29,1.01) | 0.054 |
| **Cesarean section** |  |  |  |  |
| Yes | 1 |  | 1 |  |
| No | 1.48(1.04,2.10) | 0.030 | 1.32(0.93,1.88) | 0.122 |
| **Gender** |  |  |  |  |
| Boy | 1.3(1.02,1.47) | 0.030 | 1.26(0.92,1.75) | 0.155 |
| Girl | 1 |  | 1 |  |
| **ANC visits** |  |  |  |  |
| Less than 4 | 1 |  | 1 |  |
| Above 4 | 1.01(0.80,1.51) | 0.560 | 1.20(0.77,1.83) | 0.401 |
| Don’t know | 1.22(0.61,2.46) | 0.580 | 1.51(0.67,3.38) | 0.320 |
| **Division** |  |  |  |  |
| Barisal | 0.81(0.54,1.22) | 0.309 | 1.18(0.57,2.44) | 0.663 |
| Chittagong | 0.88(0.63,1.22) | 0.428 | 1.14(0.58,2.22) | 0.709 |
| Dhaka | 0.76(0.54,1.08) | 0.123 | 1.32(0.71,2.46) | 0.388 |
| Khulna | 0.86(0.58,1.27) | 0.446 | 1.05(0.54,2.04) | 0.898 |
| Mymensingh | 0.90(0.59,1.37) | 0.612 | 1.28(0.51,3.24) | 0.603 |
| Rajshahi | 1.03(0.72,1.48) | 0.854 | 1.24(0.62,2.47) | 0.547 |
| Sylhet | 1.45(0.97,2.16) | 0.071 | 1.66(0.65,4.25) | 0.287 |
| Rangpur | 1 |  | 1 |  |
| **Women education level** |  |  |  |  |
| Primary or secondary | 1.73(1.27,2.34) | <0.001 | 1.86(1.15,2.98) | 0.011 |
| Higher secondary | 1 |  | 1 |  |
| **Birth order** |  |  |  |  |
| 1st parity | 1.29(1.07,1.55) | <0.001 | 1.10(0.75,1.64) | 0.617 |
| 2nd parity | 1 |  | 1 |  |
| 3rd parity | 1.42(1.08,1.88) | 0.010 | 1.96(0.95,4.02) | 0.068 |
| **Women Age** |  |  |  |  |
| 15-19 | 1.61(1.09,2.37) | 0.010 | 1.36(0.60,3.09) | 0.458 |
| 20-24 | 1.68(1.25,2.27) | <0.001 | 1.90(0.93,3.87) | 0.078 |
| 25-29 | 1.50(1.11,2.02) | 0.010 | 1.62(0.86,3.04) | 0.135 |
| 30-34 | 1 |  | 1 |  |
| 35-39 | 1.34(0.90,1.99) | 0.140 | 1.08(0.46,2.52) | 0.855 |
| 40-44 | 1.76(0.95,3.25) | 0.070 | 2.37(0.45,12.56) | 0.311 |
| 45-49 | 1.95(0.63,6.05) | 0.250 | 4.60(0.99,21.29) | 0.051 |
| **Wealth index** |  |  |  |  |
| Poorest | 1.71(1.25,2.33) | 0.010 | 1.78(0.94,3.40) | 0.079 |
| Middle | 1.39(1.01,1.90) | 0.040 | 1.59(0.99,2.56) | 0.054 |
| Richest | 1 |  | 1 |  |

* **IRR = Incidence Rate Ratio**
