# Supplementary material for: A study of association between maternal tetanus toxoid immunization and neonatal mortality in the context of Bangladesh
Source: PLoS One. 2025 Jan 17;20(1):e0316939. doi: 10.1371/journal.pone.0316939 (PMC11741588; doi:10.1371/journal.pone.0316939)
Supplement: Attachment — Submitted filename: PONE-D-24-08202_R2 - MTT in Bangladesh.pdf [file pone.0316939.s005.pdf]

PLOS ONE

A Study of Association Between Maternal Tetanus Toxoid Immunization and Neonatal Mortality in the Context of Bangladesh

--Manuscript Draft--

**Manuscript Number:**	PONE-D-24-08202R2
**Article Type:**	Research Article
**Full Title:**	A Study of Association Between Maternal Tetanus Toxoid Immunization and Neonatal Mortality in the Context of Bangladesh
**Short Title:**	Association Between Maternal Tetanus Toxoid Immunization and Neonatal Mortality in the Context of Bangladesh
**Corresponding Author:**	Md Jamal Uddin, Ph.D<br>Shahjalal University of Science and Technology (SUST)<br>Sylhet, BANGLADESH
**Keywords:**	tetanus; tetanus toxoid vaccine; Neonatal death; Neonatal mortality; maternal tetanus; adequate vaccination
**Abstract:**	Background<br><br>Maternal tetanus toxoid (MTT) immunization during pregnancy remains a substantial and treatable factor contributing to infant mortality globally, especially in developing nations including Bangladesh. Despite commendable progress in reducing child mortality through widespread MTT vaccination during pregnancy, the issue persists. This study explores the impact of MTT vaccination on neonatal mortality in Bangladesh and identifies associated factors.<br><br>Methods<br><br>This research utilizes data from the 2019 Bangladesh Multiple Indicator Cluster Survey (MICS). The Poisson model was employed to evaluate the influence of maternal tetanus vaccination on infant mortality with the outcome variable being the Death of a neonatal child and the independent variables considered as potential factors for the cause of death being the tetanus toxoid vaccination status, birth by cesarean section or not, mother's education level, etc.<br><br>Results<br><br>The 2019 MICS survey, covering 23,402 neonatal births, reported a 2.51% neonatal mortality rate. Notably, 45.90% of mothers received MTT vaccination during pregnancy. Among these, 23.07% received a single dose and 22.82% took adequate doses (receiving more than two doses), and adhered to WHO guidelines. Neonates born to mothers without tetanus toxoid doses had a 36.4% higher neonatal mortality rate (IRR = 1.36, p = 0.081), emphasizing the significant influence of maternal immunization. Even after adjusting for confounding factors (Crude IRR = 1.19, p = 0.181), the association remained noteworthy.<br><br>Conclusion<br><br>Maternal Tetanus Toxoid immunization plays a crucial role in reducing child mortality. The study underscores various contributors to neonatal mortality, encompassing maternal healthcare, vaccination, delivery procedures, socio-economic status, and education. Targeted interventions addressing these factors have the potential to efficiently decrease neonatal mortality rates and improve overall maternal and child health.
**Order of Authors:**	Md Jamal Uddin, Ph.D
	Sujan Kumar Naha, B.sc
	Md. Efty Islam Arpon, B.sc

Powered by Editorial Manager® and ProduXion Manager® from Aries Systems Corporation

	Rifa Tasfia Siddique, B.sc
	Farjana Rahman Ripa, B.sc
	Mohammad Nayeem Hasan, M.Sc
**Response to Reviewers:**	Thank you for your invitation to submit a revised version of our manuscript "Maternal Tetanus Toxoid Immunization and Neonatal Mortality in the Context of Bangladesh: a study of association between neonatal mortality and tetanus toxoid vaccination" (PONE-D-24-08202R1).<br>According to the comments made by the respected reviewers we modified our manuscript. We look forward to your positive response.<br><br>Sincerely,<br><br>On behalf of all authors,<br>Dr. Md Jamal Uddin (Corresponding authors)<br>Professor of Biostatistics and Epidemiology,<br>Department of Statistics,<br>Shahjalal University of Science and Technology,<br>Sylhet, Bangladesh.<br>Phone: +8801716972846<br>Email: jamal-sta@sust.edu
**Additional Information:**	
**Question**	**Response**
**Financial Disclosure**<br><br>Enter a financial disclosure statement that describes the sources of funding for the work included in this submission. Review the submission guidelines for detailed requirements. View published research articles from *PLOS ONE* for specific examples.<br><br>This statement is required for submission and **will appear in the published article** if the submission is accepted. Please make sure it is accurate.<br><br>**Funded studies**<br>Enter a statement with the following details:<br>• Initials of the authors who received each award<br>• Grant numbers awarded to each author<br>• The full name of each funder<br>• URL of each funder website<br>• Did the sponsors or funders play any role in the study design, data collection and analysis, decision to publish, or preparation of the manuscript?<br><br>Did you receive funding for this work?	The author(s) received no specific funding for this work.

**Competing Interests**<br><br>Use the instructions below to enter a competing interest statement for this submission. On behalf of all authors, disclose any competing interests that could be perceived to bias this work—acknowledging all financial support and any other relevant financial or non-financial competing interests.<br><br>This statement is required for submission and **will appear in the published article** if the submission is accepted. Please make sure it is accurate and that any funding sources listed in your Funding Information later in the submission form are also declared in your Financial Disclosure statement.<br><br>View published research articles from *PLOS ONE* for specific examples.<br><br>**NO authors have competing interests**<br><br>Enter: *The authors have declared that no competing interests exist.*<br><br>**Authors with competing interests**<br><br>Enter competing interest details beginning with this statement:<br><br>*I have read the journal's policy and the authors of this manuscript have the following competing interests: [insert competing interests here]*<br><br>* typeset	No competing interests exist for this manuscript.
**Ethics Statement**<br><br>Enter an ethics statement for this submission. This statement is required if the study involved:<br><br>• Human participants<br>• Human specimens or tissue<br>• Vertebrate animals or cephalopods	This study solely utilized pre-existing, anonymized data from the 2019 MICS survey, no further ethical approval was required for this specific analysis.

Powered by Editorial Manager® and ProduXion Manager® from Aries Systems Corporation

• Vertebrate embryos or tissues<br>• Field research<br><br>Write "N/A" if the submission does not require an ethics statement.<br><br>General guidance is provided below. Consult the submission guidelines for detailed instructions. **Make sure that all information entered here is included in the Methods section of the manuscript.**	

**Format for specific study types**<br><br>**Human Subject Research (involving human participants and/or tissue)**<br>• Give the name of the institutional review board or ethics committee that approved the study<br>• Include the approval number and/or a statement indicating approval of this research<br>• Indicate the form of consent obtained (written/oral) or the reason that consent was not obtained (e.g. the data were analyzed anonymously)<br><br>**Animal Research (involving vertebrate animals, embryos or tissues)**<br>• Provide the name of the Institutional Animal Care and Use Committee (IACUC) or other relevant ethics board that reviewed the study protocol, and indicate whether they approved this research or granted a formal waiver of ethical approval<br>• Include an approval number if one was obtained<br>• If the study involved *non-human primates*, add *additional details* about animal welfare and steps taken to ameliorate suffering<br>• If anesthesia, euthanasia, or any kind of animal sacrifice is part of the study, include briefly which substances and/or methods were applied<br><br>**Field Research**<br><br>Include the following details if this study involves the collection of plant, animal, or other materials from a natural setting:<br>• Field permit number<br>• Name of the institution or relevant body that granted permission	
**Data Availability**<br><br>Authors are required to make all data underlying the findings described fully available, without restriction, and from the time of publication. PLOS allows rare exceptions to address legal and ethical concerns. See the PLOS Data Policy and FAQ for detailed information.	Yes - all data are fully available without restriction

Powered by Editorial Manager® and ProduXion Manager® from Aries Systems Corporation

A Data Availability Statement describing where the data can be found is required at submission. Your answers to this question constitute the Data Availability Statement and **will be published in the article**, if accepted.<br><br>**Important:** Stating 'data available on request from the author' is not sufficient. If your data are only available upon request, select 'No' for the first question and explain your exceptional situation in the text box.<br><br>Do the authors confirm that all data underlying the findings described in their manuscript are fully available without restriction?	
**Describe where the data may be found in full sentences. If you are copying our sample text, replace any instances of XXX with the appropriate details.**<br><br>• If the data are **held or will be held in a public repository**, include URLs, accession numbers or DOIs. If this information will only be available after acceptance, indicate this by ticking the box below. For example: *All XXX files are available from the XXX database (accession number(s) XXX, XXX.).*<br>• If the data are all contained **within the manuscript and/or Supporting Information files**, enter the following: *All relevant data are within the manuscript and its Supporting Information files.*<br>• If neither of these applies but you are able to provide **details of access elsewhere**, with or without limitations, please do so. For example:<br><br>*Data cannot be shared publicly because of [XXX]. Data are available from the XXX Institutional Data Access / Ethics Committee (contact via XXX) for researchers who meet the criteria for access to confidential data.*<br><br>*The data underlying the results presented in the study are available from (include the name of the third party*	All data files are available from the figshare database.<br>DOI : (https://doi.org/10.6084/m9.figshare.27210603)

and contact information or URL).<br>• This text is appropriate if the data are owned by a third party and authors do not have permission to share the data.<br><br>* typeset	
Additional data availability information:	Tick here if the URLs/accession numbers/DOIs will be available only after acceptance of the manuscript for publication so that we can ensure their inclusion before publication.

Powered by Editorial Manager® and ProduXion Manager® from Aries Systems Corporation

Manuscript
Click here to access/download;Manuscript;Manuscript file_REVISED_Cleaned.docx

A Study of Association Between Maternal Tetanus Toxoid Immunization and Neonatal Mortality in the Context of Bangladesh

Sujan Kumar Naha[1*], Md. Efty Islam Arpon[1*], Rifa Tasfia Siddique[1], Farjana Rahman Ripa[1], Mohammad Nayeem Hasan[1], Md Jamal Uddin[1,2**]

[1]Department of Statistics, Shahjalal University of Science & Technology, Sylhet-3114, Bangladesh.
[2]Department of Graduate Studies, Daffodil International University, Dhaka, Bangladesh
*Equal contributions
Corresponding Author: Md Jamal Uddin; jamal-sta@sust.edu; Department of Statistics, Shahjalal University of Science and Technology, Sylhet-3114, Bangladesh.

Abstract

Background: Maternal tetanus toxoid (MTT) vaccination during pregnancy remains an important factor for reducing infant mortality globally, especially in developing nations, including Bangladesh. Despite commendable progress in reducing child mortality through widespread MTT vaccination during pregnancy, the issue still exists. This analysis explores the impact of MTT vaccination on neonatal mortality in Bangladesh and identifies associated factors.

Methods: This research utilizes data from the 2019 Bangladesh Multiple Indicator Cluster Survey (MICS). The dataset consists of 23,402 cases; among them, 587 cases resulted in infant death. The outcome variable was infant mortality, which was binary. The independent variables identified as potential contributors to the cause of death included tetanus toxoid vaccination status, mode of delivery (cesarean section or not), and mother's education level, among others. The Poisson model was employed to analyze the data.

Results: The analyses showed that the neonatal mortality rate was 2.51%. Notably, 45.90% of mothers received the MTT vaccination during pregnancy. Among them, 23.07% received a single dose, and 22.82% took adequate doses (receiving more than two doses) and adhered to WHO guidelines. The adjusted incidence rate ratio (IRR) was 1.36, which indicates that there was a 36% higher risk of neonatal mortality

for those children whose mothers did not take TT (IRR = 1.36, p = 0.081). We also found that women from middle-class households (IRR = 1.58, 95% CI = 0.98, 2.54) and women with higher parity (IRR = 1.96, 95% CI = 0.95, 4.03) also had a higher risk of newborn fatalities. A comparable trend has been observed regarding the correlation between the number of tetanus doses administered and neonatal mortality, where it also emphasizes the importance of receiving adequate doses (a minimum of 2 doses of tetanus vaccine) to mitigate neonatal mortality (adjusted IRR = 0.54, 95% CI = 0.29, 1.01) in comparison to no doses received.

Conclusion: Administering a minimum of one maternal tetanus dose significantly lowers the risk of neonatal mortality. Other than Maternal Tetanus Toxoid vaccination, the analyses underscore various contributors to neonatal mortality, encompassing maternal healthcare, delivery procedures, socio-economic status, and education. Targeted interventions addressing these factors have the potential to efficiently decrease neonatal mortality rates and improve overall maternal and child health.

Keywords: Tetanus, tetanus toxoid vaccine, neonatal death, neonatal mortality, maternal tetanus, adequate doses.

Introduction

Tetanus is an acute infectious disease that continues to pose a significant public health challenge. It occurs when the umbilical stump is covered with contaminated material or when non-sterile tools are used to cut the umbilical cord (1). It can complicate surgery, intramuscular injections, gangrene, burns, ulcers, necrotic snakebites, middle ear infections, septic abortions, and childbirth (2). Tetanus can impact individuals across all age groups; however, it presents a particularly significant risk to newborns and pregnant women lacking adequate tetanus-toxoid vaccinations. In such cases, the disease can often result in death or significant health complications (3).

The disease is caused by *Clostridium tetani* bacteria. This common bacterium is typically found in soil, but it can also be found in human and domestic animal feces (4). Spores of *Clostridium tetani* enter the human body through wounds or small abrasions when anaerobic conditions are met. Tetanus can be avoided with proper care whenever a wound arises and adequate vaccination (5). However, it should be noted that tetanus cannot be completely eliminated due to the presence of spores in the natural environment.

Maternal and neonatal tetanus (MNT) is a significant public health issue that impacts mothers and their newborns. It is prevalent when women give birth in unsanitary conditions and do not receive the complete series of tetanus toxoid (TT) vaccinations. So, an effective approach to reducing the spread of the disease is through the immunization of newborns and the women who are planning to become pregnant. This immunization can be achieved by vaccination with tetanus-toxoid-containing vaccines (TTCV). Maternal and neonatal tetanus (MNT) protection through vaccination ensures that a baby remains protected from the disease during the initial two months of life (6).

The tetanus-toxoid-containing vaccines (TTCV) are typically given to reproductive women between the ages of 15 and 44 years to protect both mother and newborn and prevent maternal and neonatal tetanus.

A woman needs five tetanus toxoid doses to be protected against tetanus for life (6). For children, the World Health Organization (WHO) recommends six doses of TTCV, three of which are primary and three of which are boosters. The three-dose primary series should start as early as six weeks of age, with the following doses spaced at least four weeks apart. Preferably, the three booster doses should be administered between the ages of 4 and 7 years, 12 and 23 months, and 9 and 15 years (7). Booster immunization campaigns are directed toward women of childbearing age, and in many nations, they have significantly decreased the incidence of tetanus in both mothers and newborns (5).

It has been challenging to enhance neonatal health in low-income countries because babies have less access to health care and are thus more at risk of worse health outcomes (8). For instance, a prior study between 1997 and 2002 discovered that 54.7% of newborn deaths happened during the neonatal period (9). A failure in routine prenatal immunization programs, below average antenatal care services, an increase in home deliveries, and unhygienic deliveries result in a mortality rate of about 35% and proximal (309000) deaths from maternal or neonatal tetanus (10). Tetanus caused an estimated 787,000 infant fatalities in 1988, with an estimated proportionate mortality rate of 6.7 per thousand live births, demonstrating the disease's significant contribution to the world's high rate of neonatal mortality (11). Globally, reported newborn tetanus mortality dropped by 85% between 2000 and 2018, from 170,829 to 25,000 deaths (12). Approximately 25,000 fewer newborns died in 2018 compared to 215,000 in 1999, a considerable decline since that year. Out of 59 low- and middle-income at-risk nations, MNT has currently been eradicated in 47 of them (13).

The Bangladesh Demographic and Health Survey reported neonatal mortality rates of 28 per 1,000 live births in 2014 and 30 in 2017, while under-five mortality rates were 46 per 1,000 in 2014 and 45 in 2017 (16,17). The current rate is significantly higher than the global average of 6.6 per 1,000 live births in 2015 and exceeds the 7.4 per 1,000 averages in developing countries and neighboring countries like India and

Nepal (18). The WHO reports that preterm birth causes 30% of global neonatal deaths, sepsis or pneumonia 27%, birth asphyxia 23%, congenital abnormalities 6%, neonatal tetanus 4%, diarrhea 3%, and other causes 7% (19–21). An exploratory study in rural Bangladesh found that tetanus accounts for 42% of newborn deaths (22). According to another study, out of 330 infant deaths, 112 of them fit the tetanus case description (23). A study in 2016 found that there were 24.4 neonatal deaths for every 1000 live births in Bangladesh, based on 6748 neonatal fatalities spread throughout four districts (Thakurgaon, Jamalpur, Moulvibazar, and Narail), with a combined population of 6.7 million (24).

Tetanus is still prevalent and a major cause of death in low-income countries, despite considerable World Health Organization programs that effectively target maternal and newborn disease (14). As opposed to low-income countries (LICs), High-Income Countries (HICs) like the United States of America, England, and Denmark have long since managed tetanus-related newborn mortality (15). A few Low and Middle-Income Countries (LMICs), like Bangladesh, Afghanistan, and Nepal, are also making progress in lowering neonatal tetanus fatality rates (11) even though the pace of reduction is very low (16).

Effective intervention programs have been implemented in these countries, which include improved cord care, increased tetanus toxoid coverage for pregnant women, adoption of safe birth practices, and implementation of postpartum care procedures. Despite some successes in countries such as Bangladesh and Nepal, low- and middle-income countries (LMICs) still face challenges in reducing infant mortality caused by neonatal tetanus. According to our knowledge, no study ever before was able to give a clear picture of the influence of maternal tetanus toxoid vaccination in reducing neonatal mortality in Bangladesh. The objective of our study was to investigate the potential link between neonatal mortality and tetanus toxoid immunization among women aged 15-49 in Bangladesh. This study will assist policymakers and planners in making more informed and effective decisions regarding this important issue.

Materials and methods

Description of the dataset:

The research was conducted using a cross-sectional study design, utilizing the accessible data from the Bangladesh Multiple Indicator Cluster Survey (MICS) conducted by the Bangladesh Bureau of Statistics (BBS) in collaboration with the United Nations International Children's Emergency Fund (UNICEF) in 2019. The survey was carried out from January 19 to June 1, 2019. The survey sample was selected through a two-stage, stratified cluster sampling method in order to recruit the participants. The 2011 Bangladesh Census of Population and Housing was used as the sampling frame for this study. The enumeration areas (EAs) selected for the census enumeration were the primary sampling units (PSUs) selected during the initial stage. In each sample, a household listing was completed as part of the research process. In the second stage, a sample of households was selected for further analysis. The sample was created to generate estimates for various variables concerning women and children across the entire country, including both urban and rural areas in all 64 districts of the seven administrative divisions. The survey consisted of a sample of 64,400 households and approximately 3,220 primary sampling units (PSUs) (25). The households were thoroughly interviewed by trained interviewers using questionnaires that covered reproductive history, prenatal and postnatal care, family planning, and demographic characteristics. The datasets were made available to the public for access and were specifically collected from sampling units (PSUs) (26).

Children who have passed away during the neonatal period or within the first month after birth may be considered as eligible cases for participation in the study. Moreover, it is important to note that the study subjects were specifically chosen to include children who had a high risk of newborn mortality, even though they were still alive at the time of the survey. Births that occurred prior to 2014 or five years before the survey are not classified as cases.

Study Variables

The outcome variable is the child's survival status in the neonatal stage, which is binary and classified as either 0 (for alive) or 1 (for death). The analysis utilized data on the age at which mortality events occurred among live births that took place in the five-year period prior to the MICS 2019 survey. The variable indicating whether or not a neonatal death occurred was recoded using the standard procedures outlined in the SPSS syntax files provided by UNICEF (27). These recoded data were used to create the outcome variable for the research analysis.

The principal factor under investigation as a potential exposure variable was the administration of the tetanus toxoid vaccine, classified as "0" for non-taken or "1" for taking at least 1 dose. Again, at least two doses are considered adequate according to the WHO. In addition to the principle factor, the additional modifiable risk factors analyzed in this research consisted of cesarean section (yes, no), gender of the neonate (boy and girl), antenatal care visits (less than 4, 4 and above), division (Barisal, Chittagong, Dhaka, Khulna, Rajshahi, Rangpur, and Sylhet), mother's education level (primary, higher secondary), birth order (1, 2, and 3), mother's age during pregnancy (15-20, 20-24, 25-29, 30-34, 35-39, 40-44, and 45-49), socioeconomic status (poorest, middle, and richest).

Statistical Analysis

Chi-square tests have been implemented to assess the relationship between potential exposure or control variables and infant mortality, forming the foundation for our models. Factors in bivariate models with p-value < 0.2 were included in the multivariable model, along with a previous multicollinearity test, and this threshold for p-value has been taken from a previously published paper (28,29).

For highly skewed binary data with significant class imbalance, Poisson regression can be preferable over logistic regression. Poisson regression performs better because of its log transformation, which

improves results stability and interpretability. This approach is particularly advantageous in cases of high skewness, as it offers reliable estimates and reduces the risk of overfitting. The Poisson model effectively estimates relative risk and manages variance overestimation, making it a robust choice for binary outcomes in such contexts (30,31). Our data's extreme imbalance and skewness align well with Poisson regression’s strengths. Therefore, the final analysis was carried out using a Poisson regression model to examine the relationship between the outcome variable and several predictor variables, since in our data the proportion of death is less than 5% (approximately 2.54%), indicating a high skewness in child mortality. The model included all variables deemed significant by chi-square analysis, ensuring a comprehensive examination of their impact on child mortality. The missing values were removed with case-wise deletion as per the default data handling procedure in STATA. For each predictor variable, the incidence rate ratio (IRR) and the 95% confidence interval (CI) for child deaths were calculated.

Ethics Statement

The analysis utilized publicly accessible datasets from health surveys, ensuring that all identifiable personal information was removed. The analysis conducted in this study utilized secondary data sources, thereby exempting it from the need for ethical review approval from the relevant institutions. Nonetheless, the methods employed in MICS underwent a thorough review and received approval from both UNICEF and BBS.

Results

The most recent MICS (2019) survey data were integrated into this study. Among the 23,402 neonatal births, there were 587 neonatal fatalities, accounting for approximately 2.54% of the total neonatal births (Fig. 1). Approximately 45.9% of women received at least one dose of tetanus toxoid immunization during their most recent pregnancy, while 22.8% of women received the adequate number of doses. A multitude of factors played a role in the occurrence of early newborn deaths. In the univariate analysis presented in Table 1, it was observed that 67.2% of women underwent cesarean section, while the percentage of normal deliveries stood at 32.8%. Notably, the death rate associated with cesarean sections was low, recorded at 1.7%. Observations indicate that neonatal mortality rates are elevated in boys at 2.7%, compared to 2.3% in girls during delivery. It is worth mentioning that the neonatal mortality rates were 1.7% for mothers who had fewer than four antenatal care (ANC) visits. On the other hand, mothers who had four or more ANC visits had a slightly lower neonatal mortality rate of 1.21%. Furthermore, a minor percentage of mothers (0.13%) reported uncertainty regarding the number of antenatal care visits they had attended. Notably, a substantial correlation in univariate analysis has been identified between division and neonatal mortality, with mothers from the Sylhet division experiencing the highest neonatal mortality rate at 3.7%. It is important to highlight that education significantly influences neonatal mortality rates. Women with primary or secondary education experienced a 2.7% rate of neonate deaths, while those with higher secondary education had a rate of 1.6%. Birth order appears to be an important factor, as second-parity births are linked to increased neonatal mortality, highlighting the need for further investigation into the underlying causes. Women with increased birth parity experience elevated neonatal mortality, which constitutes another critical factor. The wealth index of the respondents indicates that 45.6% came from poor families, 37.2% were from the middle class, and 16.7% belonged to the rich category. The neonatal mortality rates are observed to be 2.9% for the poorest, 2.4% for the middle class, and 1.7% for the richest segments of the population. Finally, it is noteworthy that

a considerable proportion of women aged 20–24 experienced a significant rate of newborn infant mortality, recorded at 2.9%.

Fig 1. Proportion of dead and alive neonates

Table 1 displays the mortality status row proportions across different covariate categories. It is evident from the table that the respondents classified in the lowest wealth index category experience the highest percentage of neonatal deaths at 2.9%, in contrast to the richest group at 1.7% and the middle class at 2.4%. The Sylhet division exhibits the highest proportion of neonatal mortality at 3.7%, closely followed by the Rajshahi division at 2.9%. The mortality rate is notably elevated in individuals with a third party or higher, recorded at 3.1%. Additionally, mothers who experienced natural births exhibit a greater percentage of neonatal deaths (4.8%) in contrast to those who underwent cesarean sections (3.1%). Finally, the mothers who received the tetanus toxoid vaccine exhibited a lower neonatal mortality rate of 3.3% compared to the 2.9% observed in non-vaccinated mothers during their last pregnancy.

[Table 1 Here]

From the figures, we get an insight into the association and distribution among some variates. The second figure (Fig. 2) highlights the importance of tetanus vaccination in reducing neonatal deaths. Regions with higher vaccination rates among neonates, such as Barishal, show lower percentages of neonatal deaths. Conversely, areas like Sylhet, which have lower immunization rates, have a greater incidence of infant fatalities. This pattern underscores the critical role of tetanus vaccination in improving neonatal survival rates, as evidenced by the significantly lower mortality rates in areas with better vaccination coverage. (Fig.

3) suggests that first-parity births have the highest rate of vaccination, and with subsequent births, the proportion of vaccinated individuals decreases. Relating this to Table 1, it is observed that the 3rd or higher parity births also have a higher proportion of neonatal deaths, implying the importance of vaccination. (Fig. 4) represents the proportion of doses of the tetanus toxoid vaccine taken by the mother where the newborn faced a neonatal death. Further implying the significance of adequate tetanus toxoid vaccination, it is observed that, among the neonatal deaths, a significantly large proportion of mothers (56.8%) had taken no tetanus toxoid vaccine during pregnancy, and only 19% of the mothers had an adequate amount of at least 2 doses of tetanus toxoid vaccine.

Fig 2. Tetanus toxoid vaccination status across different divisions

Fig 3. Tetanus toxoid vaccination status among different birth order

Fig 4. Tetanus Toxoid Vaccination Among Mothers with Neonatal Mortality

The results of the regression analysis of the factors that predict newborn mortality are shown in Table 2. In the Poisson regression model, including the variables with $p < 0.2$, $p < 0.1$, $p < 0.05$, $p < 0.01$, $p < 0.001$ in the univariate analysis, tetanus doses, women's education level, women with a higher index wealth quintile, and women with a higher parity were found to be significantly linked with newborn mortality in the Poisson regression model. Crude incidence rate ratios (IRRs) for death are shown in the characteristics table for women who had tetanus toxoid (TT) immunization vs. those who did not. When compared to the vaccinated group, the unvaccinated group's crude IRR for death was 1.19 at first, with a p-value of 0.18, indicating no discernible difference in mortality. However, after adjusting for the other factors in the model, the crude IRR rose to 1.36 with a p-value of 0.08. Although this is not statistically significant at the traditional cutoff point of $p < 0.05$, this adjusted IRR suggests an elevated risk of MTT on infant mortality. According to our analysis,

mothers with no tetanus dose had a 36% increased incidence rate ratio of experiencing newborn mortality (IRR = 1.36, 95%CI = 0.96, 1.93) compared to mothers taking at least a single tetanus dose. A similar trend has been found in the relationship between the number of tetanus doses taken and neonatal mortality that has been given in Appendix 1, which also describes taking adequate doses (at least 2 doses of tetanus vaccine) to reduce neonatal mortality (Crude IRR = 0.87, 95%CI = 0.57, 1.32) compared to none taken host. Interestingly, the cesarean section was found insignificant. We also discovered that women's education level was significantly associated with newborn mortality, mothers with primary or secondary education had an increased 86% incidence rate ratio of newborn mortality (IRR = 1.86, 95%CI = 1.16, 2.99) compared to mothers who had studied at the higher secondary level. Additionally, women from poor families had a significantly higher incident rate ratio compared to the women from rich families (IRR = 1.77, 95%CI = 0.93, 3.38). Similarly, women from middle-class families had a significantly higher incident rate ratio compared to the women from rich families (IRR = 1.58, 95%CI = 0.98, 2.54). In addition, third-parity births were found to be substantially correlated with newborn mortality when compared to the second-parity births (IRR = 1.96, 95%CI = 0.95, 4.03). This indicates that women with higher parity had a 96% increased risk of neonatal deaths. Women aged between 25 and 29 years indicated a fairly significant association (IRR = 1.89, p = 0.081) with newborn death, showing an 89% higher incident rate ratio of neonatal mortality compared to women aged between 30 and 35 years. Likewise, women aged between 45 and 49 years had a significantly (IRR = 4.05, p = 0.055) higher rate ratio than women aged between 30 and 35 years. Gender, antenatal care visits, and division, which are the necessary factors, were found to be insignificant in the model.

[Table 2 here]

Discussion

Tetanus demonstrates a significant threat to human life (32). Despite significant advancements over the past twenty years in reducing global tetanus-related fatalities, neonatal tetanus has garnered considerable focus, while maternal tetanus remains largely underexplored (15,33). This survey aims to identify the impact of neonatal mortality in relation to tetanus toxoid vaccination among pregnant women aged 15 to 49 years. The findings indicate that maternal vaccination plays a significant role in decreasing neonatal death rates.

Previous investigations have not explored the relationship between neonatal mortality and tetanus toxoid immunization in conjunction with other essential covariates. Our primary results demonstrate that women who obtain at least one dose of the tetanus vaccine substantially reduce the likelihood of neonatal death in their children. Furthermore, around 2.54% of neonatal deaths in Bangladesh are associated with pregnant women who did not receive at least one dose of the tetanus toxoid vaccination. In contrast, about 45.9% of women have received at least one dose of the vaccine, which could serve as valuable data for initiatives aimed at enhancing vaccination rates.

Our findings show that only 22.82% of pregnant women had received a sufficient amount of the TT vaccine, which is low as compared to a study showing that sufficient TT immunization among pregnant women was 75% worldwide (34). These findings are consistent with previous research's findings, which showed a lower prevalence of obtaining the adequate TT vaccine (23) and the rate of newborn death was 63.8 per 1000 live births (35). The prevalence of TT immunization was 81.8% for MICS (2006) and 61.3% for MICS (2012–2013) (36) and we observed in our research that for MICS (2019), the rate of taking tetanus doses was 54.1%. These findings shed light on the varying levels of vaccination coverage among pregnant women, emphasizing the need to promote and ensure full compliance with the WHO's recommended vaccination regimen during pregnancy for the optimal prevention of maternal and neonatal tetanus. Overall, in Bangladesh, the prevalence has generally been decreasing over the course of the survey years.

In our model, we discovered that tetanus doses taken, women's education level, wealth index, and birth order were significantly associated with higher odds of neonatal mortality. Our analysis shows that a mother with no tetanus doses has 36.4% more chance to experience neonatal death (IRR = 1.36, 95%CI = 0.96, 1.93) compared to a mother with at least 1 dose. Similar to other studies, we discovered that the TT vaccine had a protective effect against infant mortality when compared to IFA (iron–folic acid) supplementation alone (37–39) The evidence from India aligns with our analysis, indicating that receiving at least one dose of the TT vaccination could decrease infant death (40). Another study revealed a sharp reduction in newborn mortality as a result of a decrease in neonatal tetanus deaths (41). Tetanus vaccination coverage plays a vital role in reducing neonatal tetanus. We are the first to show the association between tetanus toxoid vaccination and neonatal mortality in Bangladesh in recent years.

Cesarean section is a potential factor for neonatal mortality according to previous studies, where cesarean section delivery is highly correlated with neonatal mortality in low-income countries like Bangladesh (8,42). However, we found no association between neonatal mortality and cesarean sections, which may be a noticeable gap in our study that could be addressed with the confirmation of further research.

A mother's education significantly plays a vital role in neonatal mortality. Women who only completed elementary or secondary school had a greater risk of newborn mortality compared to women who pursued higher education. Considering that educated women may be better empowered to make decisions about their health, education may increase their understanding of the harmful impacts of tetanus and neonatal death (36,43).

Another important factor associated with neonatal mortality is higher parity. Our study revealed that women with higher parity have lower odds of neonatal mortality compared to those with lower parity. This is supported by findings from previous studies that have found higher parity to be a significant predictor of neonatal mortality (44–46). The past birth experiences of the ladies may be the cause. It's also reasonable

that women with more children are less likely to be employed or to have greater levels of education. There are further studies that show older women in the nation are less likely than younger women to use prenatal and delivery care, which can be another identifiable reason (47–49).

Various age groups in the reproductive age of women play a crucial role in reducing neonatal death. Older women (age 45-49 with IRR = 4.05, 95%CI = 0.97, 16.95) are more exposed to neonatal death than any other reproductive period, and it is one of the important issues that needs to be taken care of. Similar studies show that maternal periods in different age groups directly influence neonatal mortality, which is likely due to complications of delivery that arise at a later age (50,51).

The health of the Bangladeshi population has significantly improved over the past 20 years, and Bangladesh has been acknowledged as an example of "good health at low cost" (52). As far as we are aware, socioeconomic status has a big impact and is highly correlated; babies from the "poorest" homes are more likely to die, which emphasizes how critical it is to solve socioeconomic gaps in access to healthcare. Our research says women with wealthy households are less likely to face neonatal mortality compared to women with a poor wealth quantile index. This result is in line with earlier research demonstrating that increasing the wealth index of women living at home protects against tetanus in comparison to a low wealth index (40,43,53,54) We believe that women from wealthy families are more likely to have access to healthcare services than women from low-income families. A policy and programming requirement to ensure that all pregnant women receive at least one dose of prenatal medication, especially those in need or marginalized population subgroups, is expected to have a significant influence on improving neonatal survival. This specific intervention should be within the grasp of the public health system as an immediate priority due to difficulties in providing the entire health system functionality needed for comprehensive and safe mother and newborn care (36).

Limitations and Strengths

Strengths

To our knowledge, this study is the first in Bangladesh to investigate the relationship between maternal tetanus toxoid vaccination and neonatal mortality. We employed appropriate data analysis techniques, taking into consideration all intricate survey designs. Findings from this work can be used to inform future research, policy, and clinical practice and to benchmark progress. One of the benefits of this study is the sizeable and nationally representative sample size, and the results are applicable to the entire country. The information was gathered using the most recent survey. Our results would surely pique interest in additional research and educate decision-makers about the gaps in tetanus care that need to be filled.

Limitations

However, despite the several strengths, the existence of bias resulting from different survey time points and the cross-sectional character of the data cannot be confirmed. Some variables that were statistically insignificant but still important for the research study have been considered in the model. It is crucial to keep in mind that the mortality that is being discussed here includes deaths from all causes. Regretfully, our analysis lacks particular data that isolates the risk exclusively for newborn mortality. Notwithstanding this restriction, the trend that has been seen points to a higher risk of death for those who have not had the TT vaccination; however, statistical significance is not attained. This realization emphasizes how crucial it is to conduct additional studies to determine the specific effect of the TT vaccine on mortality outcomes, especially neonatal death. To offer more precise information, future research focusing on infant mortality rates and any confounding factors is required.

The level of significance was deemed to be fairly high. In addition, the study's drawback also emerges from the fact that we had little control over the correlated variables to include in the analysis due to the secondary data source we employed. Conclusions regarding a causal association, the relative contributions

of immunization prior versus during the most recent pregnancy, or the best possible ways to increase coverage are not possible due to the cross-sectional character of this investigation. In addition, the dependent variable for newborn death has a large number of missing values, making it impossible to include them in the analysis. This could lead to bias, and the variable varies over time, potentially changing the claimed association in longitudinal studies.

Conclusion

Premature infant death is more common among Bangladeshi women with no tetanus toxoid vaccination. Based on our findings, taking at least one maternal tetanus dose greatly reduces neonatal mortality, which clearly shows that increasing vaccination coverage as well as deploying the mandatory law of taking at least one maternal tetanus dose can save infants in great numbers. Additional investigation is required to determine the pattern of declining infant mortality in Bangladesh following vaccination against maternal tetanus toxoid since there is no evidence related to this. Furthermore, Women with higher education levels, those in the wealthiest quintile, and those with greater parity were all significantly linked to reduced odds of newborn mortality. To decrease neonatal death for the betterment of a country like Bangladesh, we advise taking help from the government and other organizations in conducting immunization campaigns, increasing vaccination coverage, and improving self-care through proper education and awareness.

Declarations

Funding
This research received no specific grant from funding agencies in the public, commercial, or not-for-profit sectors.

Conflicts of interest

The authors reported no potential conflict of interest.

Author Contributions

Conceived and designed the experiments: Md. Efty Islam Arpon, Sujan Kumar Naha. Performed the experiments: Sujan Kumar Naha, Md. Efty Islam Arpon. Analyzed the data: Sujan Kumar Naha, Mohammed Nayeem Hosaain, Md. Efty Islam Arpon. Wrote the paper: Md. Efty Islam Arpon, Sujan Kumar Naha, Rifa Tasfia Siddique, Farjana Rahman Ripa. Supervised by: Dr. Md. Jamal Uddin

The submitted article was authored by all contributors, who also provided their approval.

Acknowledgments

We acknowledge UNICEF and the Bangladesh Bureau of Statistics for allowing us to use the data.

References

1. Edmondson RS, Flowers MW. Intensive care in tetanus: management, complications, and mortality in 100 cases. BMJ [Internet]. 1979 May 26;1(6175):1401–4. Available from: https://www.bmj.com/lookup/doi/10.1136/bmj.1.6175.1401
2. Sievert DM, Rudrik JT, Patel JB, McDonald LC, Wilkins MJ, Hageman JC. Vancomycin-resistant Staphylococcus aureus in the United States, 2002-2006. Clin Infect Dis. 2008 Mar 1;46(5):668–74.
3. Thwaites CL, Beeching NJ, Newton CR. Maternal and neonatal tetanus. The Lancet. 2015 Jan;385(9965):362–70.
4. Wilkins CA, Richter MB, Hobbs WB, Whitcomb M, Bergh N, Carstens J. Occurrence of Clostridium tetani in soil and horses. S Afr Med J. 1988 Jun 18;73(12):718–20.
5. Khan MAS, Hasan MJ, Rashid MU, Kha Sagar S, Khan S, Zaman S, et al. Factors associated with in-hospital mortality of adult tetanus patients-a multicenter study from Bangladesh. PLoS Negl Trop Dis. 2022 Mar;16(3):e0010235.
6. Cook TM, Protheroe RT, Handel JM. Tetanus: a review of the literature. Br J Anaesth. 2001 Sep;87(3):477–87.
7. Tetanus vaccines: WHO position paper – February 2017 [Internet]. [cited 2024 Jun 22]. Available from: https://fctc.who.int/publications/i/item/tetanus-vaccines-who-position-paper-february-2017
8. Goldenberg RL, McClure EM. Maternal, fetal and neonatal mortality: lessons learned from historical changes in high income countries and their potential application to low-income countries. Matern Health Neonatol Perinatol. 2015;1:3.
9. Titaley CR, Dibley MJ, Agho K, Roberts CL, Hall J. Determinants of neonatal mortality in Indonesia. BMC Public Health. 2008 Jul 9;8:232.
10. Gelaw T, Ayalew S, eyene K. KNOWLEDGE AND UPTAKE OF TETANUS TOXOID VACCINE AND ASSOCIATED FACTORS AMONG REPRODUCTIVE AGE GROUP WOMEN IN HAYK TOWN SOUTH WOLLO, ETHIOPIA, CROSS-SECTIONAL STUDY. medRxiv [Internet]. 2022 Dec 21 [cited 2024 Jun 22];2022.12.20.22283731. Available from: https://www.medrxiv.org/content/10.1101/2022.12.20.22283731v1
11. Waiswa P, Kallander K, Peterson S, Tomson G, Pariyo GW. Using the three delays model to understand why newborn babies die in eastern Uganda. Trop Med Int Health. 2010 Aug;15(8):964–72.
12. Njuguna HN, Yusuf N, Raza AA, Ahmed B, Tohme RA. Progress Toward Maternal and Neonatal Tetanus Elimination - Worldwide, 2000-2018. MMWR Morb Mortal Wkly Rep. 2020 May 1;69(17):515–20.
13. Eliminating a silent killer — maternal and neonatal tetanus | UNICEF [Internet]. [cited 2024 Jun 22]. Available from: https://www.unicef.org/blog/eliminating-silent-killer-maternal-neonatal-tetanus
14. Olum S, Eyul J, Lukwiya DO, Scolding N. Tetanus in a rural low-income intensive care unit setting. Brain Commun. 2021;3(1):fcab013.
15. Khan AA, Zahidie A, Rabbani F. Interventions to reduce neonatal mortality from neonatal tetanus in low and middle income countries--a systematic review. BMC Public Health. 2013 Apr 9;13:322.
16. Dhaka A. BANGLADESH DEMOGRAPHIC AND HEALTH SURVEY 2014 National Institute of Population Research and Training Ministry of Health and Family Welfare Dhaka, Bangladesh [Internet]. 2016 [cited 2024 Jul 29]. Available from: www.mitra.bd.com.

17. NIPORT NI of PR and T, Welfare M of H and F, ICF. Bangladesh Demographic and Health Survey 2017-18 [Internet]. 2020 [cited 2024 Aug 7]. Available from: https://dhsprogram.com/publications/publication-FR344-DHS-Final-Reports.cfm
18. McAllister DA, Liu L, Shi T, Chu Y, Reed C, Burrows J, et al. Global, regional, and national estimates of pneumonia morbidity and mortality in children younger than 5 years between 2000 and 2015: a systematic analysis. Lancet Glob Health [Internet]. 2019 Jan 1 [cited 2024 Aug 7];7(1):e47–57. Available from: https://pubmed.ncbi.nlm.nih.gov/30497986/
19. Lawn JE, Cousens S, Zupan J. 4 million neonatal deaths: when? Where? Why? Lancet [Internet]. 2005 Mar 5 [cited 2024 Aug 7];365(9462):891–900. Available from: https://pubmed.ncbi.nlm.nih.gov/15752534/
20. The World Health Report 2005: Make Every Mother and Child Count - World Health Organization - Google Books [Internet]. [cited 2024 Aug 7]. Available from: https://books.google.com.bd/books?hl=en&lr=&id=4Fw0DgAAQBAJ&oi=fnd&pg=PR3&ots=5NqX5YVf-p&sig=b478n48d-bN9rsMaO7A2FfFFddU&redir_esc=y#v=onepage&q&f=false
21. Bryce J, Boschi-Pinto C, Shibuya K, Black RE. WHO estimates of the causes of death in children. Lancet [Internet]. 2005 Mar 26 [cited 2024 Aug 7];365(9465):1147–52. Available from: https://pubmed.ncbi.nlm.nih.gov/15794969/
22. Chowdhury ME, Akhter HH, Chongsuvivatwong V, Geater AF. Neonatal mortality in rural Bangladesh: an exploratory study. J Health Popul Nutr. 2005 Mar;23(1):16–24.
23. Hlady WG, Bennett J V, Samadi AR, Begum J, Hafez A, Tarafdar AI, et al. Neonatal tetanus in rural Bangladesh: risk factors and toxoid efficacy. Am J Public Health. 1992 Oct;82(10):1365–9.
24. Halim A, Dewez JE, Biswas A, Rahman F, White S, Van Den Broek N. When, Where, and Why Are Babies Dying? Neonatal Death Surveillance and Review in Bangladesh. PLoS One [Internet]. 2016 Aug 1 [cited 2024 Jun 22];11(8):e0159388. Available from: https://journals.plos.org/plosone/article?id=10.1371/journal.pone.0159388
25. Bangladesh 2019 MICS Report_English. [cited 2024 Jun 23]; Available from: https://www.unicef.org/bangladesh/media/3281/file/Bangladesh%202019%20MICS%20Report_English.pdf
26. Surveys - UNICEF MICS [Internet]. [cited 2024 Aug 7]. Available from: https://mics.unicef.org/surveys
27. Tools - UNICEF MICS [Internet]. [cited 2024 Aug 6]. Available from: https://mics.unicef.org/tools?round=mics6#analysis
28. Hasan MN, Chowdhury MAB, Jahan J, Jahan S, Ahmed NU, Uddin MJ. Cesarean delivery and early childhood diseases in Bangladesh: An analysis of Demographic and Health Survey (BDHS) and Multiple Indicator Cluster Survey (MICS). PLoS One. 2020 Dec 3;15(12):e0242864.
29. Bursac Z, Gauss CH, Williams DK, Hosmer DW. Purposeful selection of variables in logistic regression. Source Code Biol Med. 2008 Dec 16;3(1):17.
30. Cleophas TJ, Zwinderman AH. Regression Analysis in Medical Research: For Starters and 2nd Levelers. Regression Analysis in Medical Research: For Starters and 2nd Levelers. 2021 Apr 2;1–475.
31. Cleophas TJ, Zwinderman AH. Poisson Regression for Binary Outcomes (52 Patients). SPSS for Starters and 2nd Levelers [Internet]. 2016 [cited 2024 Aug 6];273–7. Available from: https://link.springer.com/chapter/10.1007/978-3-319-20600-4_47

32. Tetanus | Encyclopedia.com [Internet]. [cited 2024 Jul 29]. Available from: https://www.encyclopedia.com/medicine/diseases-and-conditions/pathology/tetanus#3435100772
33. Fauveau V, Mamdani M, Steinglass R, Koblinsky M. Maternal tetanus: Magnitude, epidemiology and potential control measures. International Journal of Gynecology & Obstetrics. 1993 Jan;40(1):3–12.
34. Health Organization W. WHO vaccine-preventable diseases: monitoring system 2009 global summary. 2009 [cited 2024 Jun 22]; Available from: www.who.int/vaccines-documents/
35. Rahman M, Chen LC, Chakraborty J, Yunus M, Chowdhury AI, Sarder AM, et al. Use of tetanus toxoid for the prevention of neonatal tetanus. 1. Reduction of neonatal mortality by immunization of non-pregnant and pregnant women in rural Bangladesh. Bull World Health Organ. 1982;60(2):261–7.
36. Amin MB, Roy N, Meem AE, Hossain E, Aktarujjaman M. Trends and determinants of taking tetanus toxoid vaccine among women during last pregnancy in Bangladesh: Country representative survey from 2006 to 2019. PLoS One. 2022;17(10):e0276417.
37. Roisin AJ, Prazuck T, Tall F, Sanou J, Cot M, Ballereau F V. Risk factor for neonatal tetanus in west Burkina Faso: a case control study. Eur J Epidemiol [Internet]. 1996 Oct;12(5):535–7. Available from: http://www.ncbi.nlm.nih.gov/pubmed/8905317
38. Gitta SN, Wabwire-Mangen F, Kitimbo D, Pariyo G, Centers for Disease Control and Prevention (CDC). Risk factors for neonatal tetanus--Busoga region, Uganda, 2002-2003. MMWR Suppl. 2006 Apr 28;55(1):25–30.
39. Chai F, Prevots DR, Wang X, Birmingham M, Zhang R. Neonatal tetanus incidence in China, 1996-2001, and risk factors for neonatal tetanus, Guangxi Province, China. Int J Epidemiol. 2004 Jun;33(3):551–7.
40. Singh A, Pallikadavath S, Ogollah R, Stones W. Maternal tetanus toxoid vaccination and neonatal mortality in rural north India. PLoS One. 2012;7(11):e48891.
41. Ronsmans C, Chowdhury ME, Alam N, Koblinsky M, El Arifeen S. Trends in stillbirths, early and late neonatal mortality in rural Bangladesh: the role of public health interventions. Paediatr Perinat Epidemiol. 2008 May;22(3):269–79.
42. Althabe F, Sosa C, Belizán JM, Gibbons L, Jacquerioz F, Bergel E. Cesarean section rates and maternal and neonatal mortality in low-, medium-, and high-income countries: an ecological study. Birth. 2006 Dec;33(4):270–7.
43. Mohamed SOO, Ahmed EM. Prevalence and determinants of antenatal tetanus vaccination in Sudan: a cross-sectional analysis of the Multiple Indicator Cluster Survey. Trop Med Health. 2022 Jan 10;50(1):7.
44. Rutstein SO. Effects of preceding birth intervals on neonatal, infant and under-five years mortality and nutritional status in developing countries: evidence from the demographic and health surveys. Int J Gynaecol Obstet. 2005 Apr;89 Suppl 1:S7-24.
45. Arokiasamy P, Gautam A. Neonatal mortality in the empowered action group states of India: trends and determinants. J Biosoc Sci. 2008 Mar;40(2):183–201.
46. Reid A. Neonatal mortality and stillbirths in early twentieth century Derbyshire, England. Popul Stud (NY). 2001 Nov;55(3):213–32.
47. Bloom SS, Lippeveld T, Wypij D. Does antenatal care make a difference to safe delivery? A study in urban Uttar Pradesh, India. Health Policy Plan. 1999 Mar;14(1):38–48.

48. Chandrashekar S, Rao RS, Nair NS, Kutty PR. Socio-demographic determinants of antenatal care. Trop Doct. 1998 Oct;28(4):206–9.
49. Ram F, Singh A. Is antenatal care effective in improving maternal health in rural uttar pradesh? Evidence from a district level household survey. J Biosoc Sci. 2006 Jul;38(4):433–48.
50. Chaudhary S, Contag S. The effect of maternal age on fetal and neonatal mortality. Journal of Perinatology 2017 37:7 [Internet]. 2017 Mar 30 [cited 2024 Aug 7];37(7):800–4. Available from: https://www.nature.com/articles/jp201736
51. Fonseca SC, Flores PVG, Camargo KR, Pinheiro RS, Coeli CM. Maternal education and age: inequalities in neonatal death. Rev Saude Publica [Internet]. 2017 [cited 2024 Aug 7];51. Available from: https://pubmed.ncbi.nlm.nih.gov/29166446/
52. Chowdhury AMR, Bhuiya A, Chowdhury ME, Rasheed S, Hussain Z, Chen LC. The Bangladesh paradox: exceptional health achievement despite economic poverty. Lancet. 2013 Nov 23;382(9906):1734–45.
53. Yaya S, Kota K, Buh A, Bishwajit G. Antenatal visits are positively associated with uptake of tetanus toxoid and intermittent preventive treatment in pregnancy in Ivory Coast. BMC Public Health. 2019 Dec 6;19(1):1467.
54. Yaya S, Kota K, Buh A, Bishwajit G. Prevalence and predictors of taking tetanus toxoid vaccine in pregnancy: a cross-sectional study of 8,722 women in Sierra Leone. BMC Public Health. 2020 Jun 5;20(1):855.

Table 1: The row-wise proportional distribution across various categories

	Neonatal mortality status		Total n (%)	P-value
	Dead n (%)	Alive n (%)		
**TT vaccinated**				
No	202(3.3)	5934(96.7)	6136(54.1)	0.255
Yes	152(2.9)	5055(97.1)	5207(45.9)	
**TT doses taken**				
None	202(3.3)	5934(96.7)	6136(54.1)	0.254
1 injection	84(3.2)	2532(96.8)	2616(23.1)	
2 injections or more (adequate doses)	68(2.6)	2519(97.4)	2587(22.8)	
**Cesarean section**				
Yes	117(3.1)	3687(96.9)	3804(67.2)	<0.001
No	90(4.8)	1769(95.2)	1859(32.8)	
**Gender**				
Boy	330(2.7)	11776(97.3)	12106(51.7)	0.028
Girl	257(2.3)	11039(97.7)	11296(48.3)	
**Antenatal care (ANC) visits**				
Less than 4	154(3.0)	5053(97.0)	5207(57.1)	0.077
Above 4	110(3.0)	3591(97.0)	3701(40.6)	
Don’t know	12(5.7)	200(94.3)	212(2.3)	
**Division**				
Barisal	49(2.4)	2020(97.6)	2069(8.8)	0.004

Chittagong	119(2.4)	4778(97.6)	4897(20.9)	
Dhaka	91(2.0)	4508(98.0)	4599(19.7)	
Khulna	70(2.2)	3158(97.8)	3228(13.8)	
Mymensingh	37(2.7)	1341(97.3)	1378(5.9)	
Rajshahi	71(2.9)	2379(97.1)	2450(10.5)	
Rangpur	76(2.7)	2689(97.3)	2765(11.8)	
Sylhet	74(3.7)	1942(96.3)	2016(8.6)	
**Women education level**				
Primary or secondary	527(2.7)	19236(97.3)	19763(84.5)	0.001
Higher secondary	60(1.6)	3579(98.4)	3639(15.6)	
**Birth order**				
1st parity	240(2.8)	8248(97.2)	8488(36.3)	
2nd parity	264(2.2)	11944(97.8)	12208(52.2)	0.002
3rd parity or more	72(3.1)	2459(96.9)	2531(10.8)	
**Women age**				
15-19	50(2.9)	1678(97.1)	1728(7.4)	
20-24	202(2.9)	6868(97.1)	7070(30.2)	0.055
25-29	170(2.5)	6711(97.5)	6881(29.4)	
30-34	91(1.9)	4707(98.1)	4798(20.5)	
35-39	55(2.5)	2188(97.5)	2243(9.6)	
40-44	16(2.9)	535(97.1)	551(2.4)	
45-49	3(2.3)	128(97.7)	131(0.6)	

**Wealth index**				
Poorest	306(2.9)	10355(97.1)	10661(45.6)	<0.001
Middle	214(2.4)	8613(97.6)	8827(37.7)	
Richest	67(1.7)	3847(98.3)	3914(16.7)	

548 **Table 2. Factor Associated with Neonatal Mortality**

Characteristics	**Crude IRR (95% CI)**	**p-value**	**Adjusted IRR (95% CI)**	**p-value**
**TT vaccinated**				
Yes	1		1	
No	1.19(0.92,1.52)	0.181	1.36(0.96,1.93)	0.081
**Cesarean section**				
Yes	1		1	
No	1.48(1.04,2.10)	0.028	1.32(0.93,1.88)	0.123
**Gender**				
Boy	1.23(1.02,1.47)	0.030	1.27(0.92,1.75)	0.151
Girl	1		1	
**ANC visits**				
Less than 4	1		1	
Above 4	1.10(0.80,1.50)	0.556	1.20(0.79,1.83)	0.395
Don't know	1.22(0.60,2.46)	0.578	1.51(0.68,3.39)	0.314
**Division**				
Barisal	0.81(0.54,1.22)	0.309	1.18(0.57,2.44)	0.659
Chittagong	0.88(0.63,1.22)	0.428	1.13(0.58,2.20)	0.729
Dhaka	0.76(0.54,1.08)	0.123	1.31(0.70,2.45)	0.392
Khulna	0.86(0.58,1.27)	0.446	1.05(0.54,2.05)	0.884
Mymensingh	0.90(0.59,1.37)	0.612	1.28(0.51,3.24)	0.599
Rajshahi	1.03(0.72,1.48)	0.854	1.25(0.63,2.49)	0.526
Sylhet	1.45(0.97,2.16)	0.071	1.66(0.65,4.25)	0.289
Rangpur	1		1	
**Women education level**				
Primary or secondary	1.73(1.27,2.34)	<0.001	1.86(1.16,2.99)	0.011
Higher secondary	1		1	
**Birth order**				

1st parity	1.29(1.07,1.56)	0.007	1.11(0.75,1.64)	0.611
2nd parity	1		1	
3rd parity	1.42(1.08,1.88)	0.014	1.96(0.95,4.03)	0.067
**Women Age**				
15-19	1.61(1.09,2.37)	0.017	1.36(0.60,3.09)	0.461
20-24	1.68(1.25,2.27)	0.001	1.89(0.93,3.86)	0.081
25-29	1.50(1.11,2.02)	0.009	1.62(0.86,3.04)	0.134
30-34	1		1	
35-39	1.34(0.90,1.99)	0.143	1.09(0.46,2.54)	0.851
40-44	1.76(0.95,3.25)	0.073	2.35(0.44,12.45)	0.317
45-49	1.95(0.63,6.05)	0.247	4.05(0.97,16.95)	0.055
**Wealth index**				
Poorest	1.70(1.25,2.33)	0.001	1.77(0.93,3.38)	0.085
Middle	1.39(1.01,1.90)	0.041	1.58(0.98,2.54)	0.060
Richest	1		1	

*** IRR = Incidence Rate Ratio**

Supplementary materials

Table 3: Analysis results for examining against Tetanus doses taken by mothers

Characteristics	Crude IRR (95% CI)	p-value	Adjusted IRR (95% CI)	p-value
**TT doses taken**				
None	1		1	
1 injection	0.84(0.64,1.09)	0.197	0.77(0.54,1.11)	0.160
2 injections or more (adequate doses)	0.87(0.57,1.32)	0.498	0.54(0.29,1.01)	0.054
**Cesarean section**				
Yes	1		1	
No	1.48(1.04,2.10)	0.030	1.32(0.93,1.88)	0.122
**Gender**				
Boy	1.3(1.02,1.47)	0.030	1.26(0.92,1.75)	0.155
Girl	1		1	
**ANC visits**				
Less than 4	1		1	
Above 4	1.01(0.80,1.51)	0.560	1.20(0.77,1.83)	0.401
Don’t know	1.22(0.61,2.46)	0.580	1.51(0.67,3.38)	0.320
**Division**				
Barisal	0.81(0.54,1.22)	0.309	1.18(0.57,2.44)	0.663
Chittagong	0.88(0.63,1.22)	0.428	1.14(0.58,2.22)	0.709
Dhaka	0.76(0.54,1.08)	0.123	1.32(0.71,2.46)	0.388
Khulna	0.86(0.58,1.27)	0.446	1.05(0.54,2.04)	0.898
Mymensingh	0.90(0.59,1.37)	0.612	1.28(0.51,3.24)	0.603

Rajshahi	1.03(0.72,1.48)	0.854	1.24(0.62,2.47)	0.547
Sylhet	1.45(0.97,2.16)	0.071	1.66(0.65,4.25)	0.287
Rangpur	1		1	
**Women education level**				
Primary or secondary	1.73(1.27,2.34)	<0.001	1.86(1.15,2.98)	0.011
Higher secondary	1		1	
**Birth order**				
1st parity	1.29(1.07,1.55)	<0.001	1.10(0.75,1.64)	0.617
2nd parity	1		1	
3rd parity	1.42(1.08,1.88)	0.010	1.96(0.95,4.02)	0.068
**Women Age**				
15-19	1.61(1.09,2.37)	0.010	1.36(0.60,3.09)	0.458
20-24	1.68(1.25,2.27)	<0.001	1.90(0.93,3.87)	0.078
25-29	1.50(1.11,2.02)	0.010	1.62(0.86,3.04)	0.135
30-34	1		1	
35-39	1.34(0.90,1.99)	0.140	1.08(0.46,2.52)	0.855
40-44	1.76(0.95,3.25)	0.070	2.37(0.45,12.56)	0.311
45-49	1.95(0.63,6.05)	0.250	4.60(0.99,21.29)	0.051
**Wealth index**				
Poorest	1.71(1.25,2.33)	0.010	1.78(0.94,3.40)	0.079
Middle	1.39(1.01,1.90)	0.040	1.59(0.99,2.56)	0.054
Richest	1		1	

*** IRR = Incidence Rate Ratio**

Figure 1

Click here to access/download;Figure;Fig 1.tiff

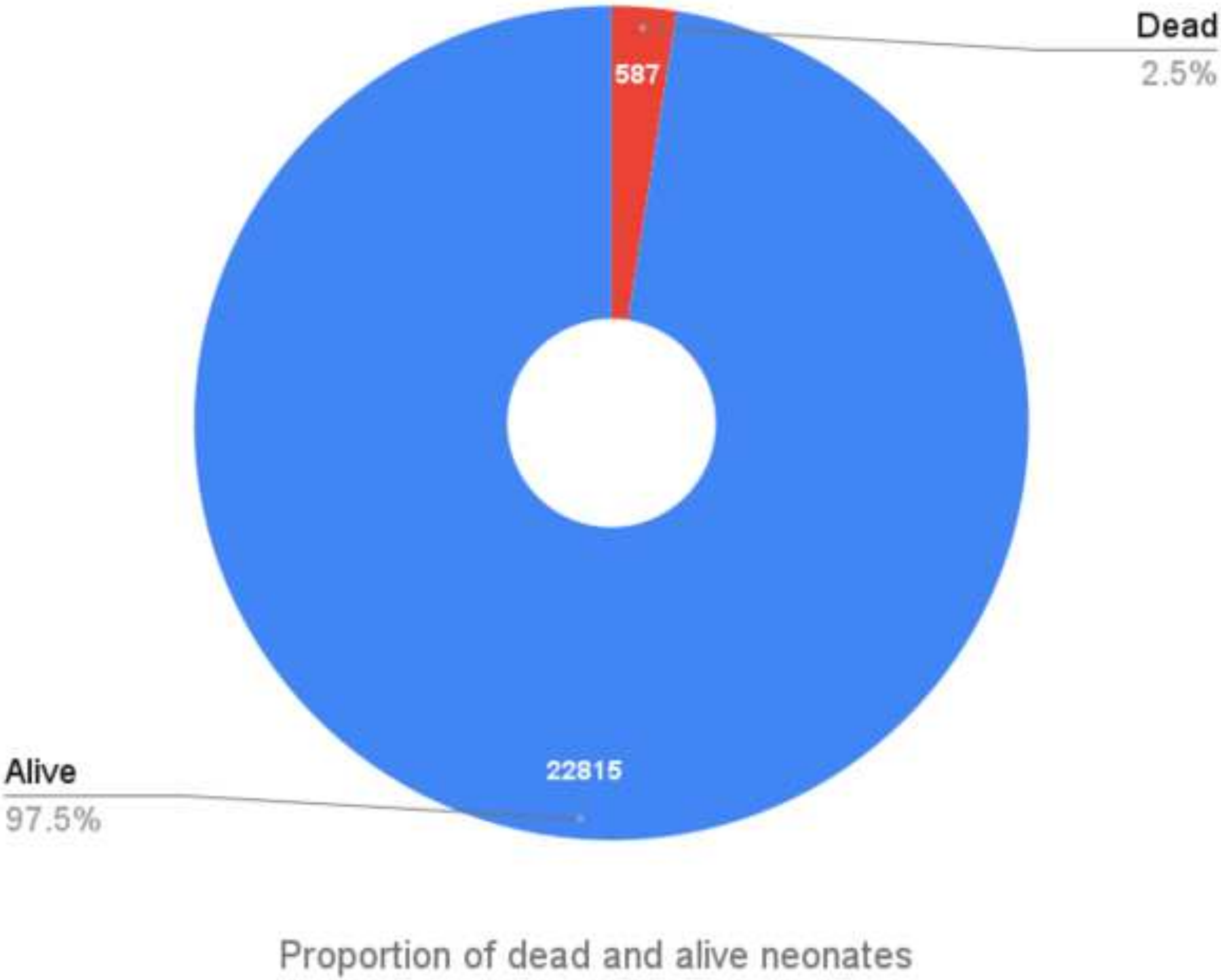

Figure 2

Click here to access/download;Figure;Fig 2.tiff

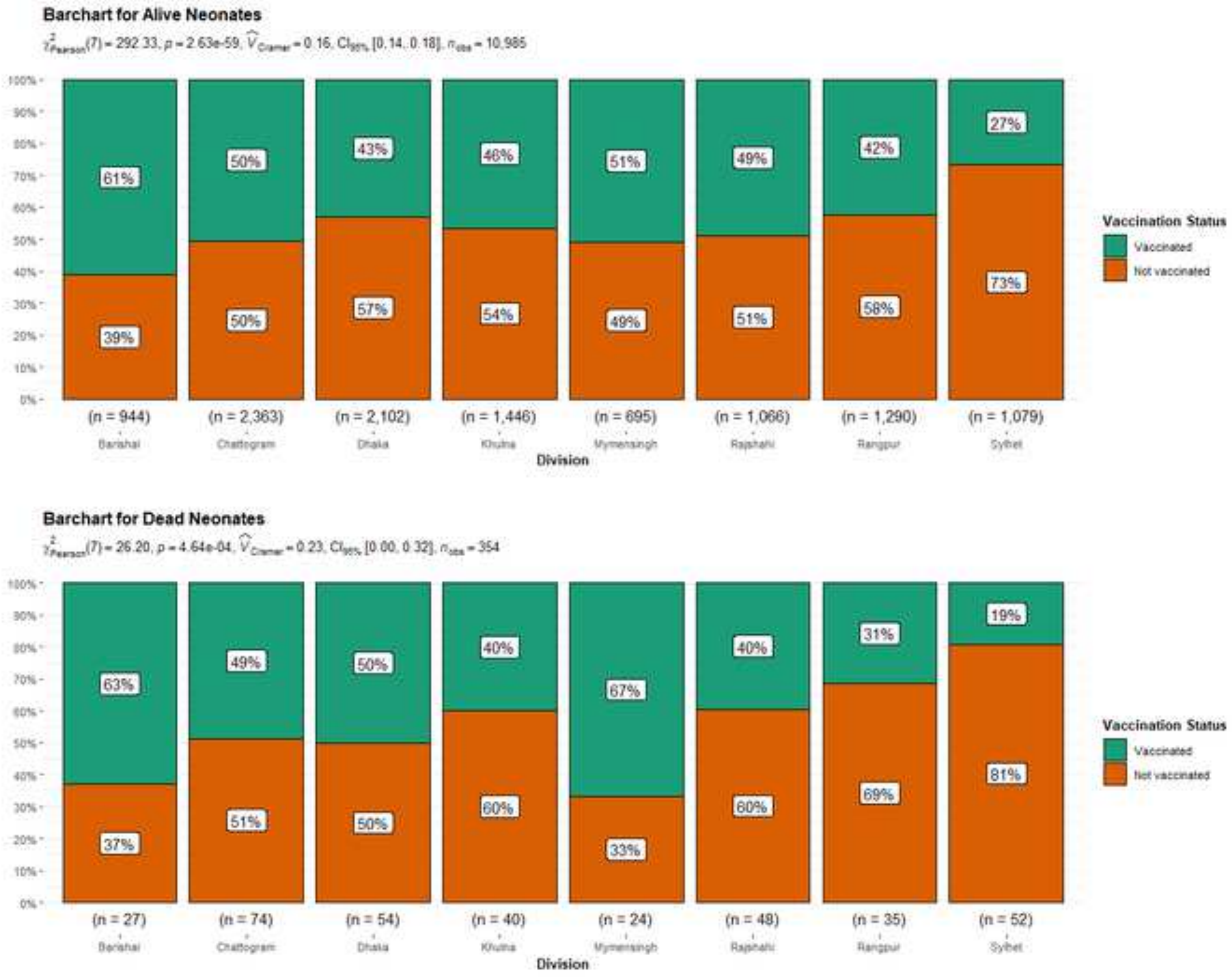

Figure 3

Click here to access/download;Figure;Fig 3.tiff

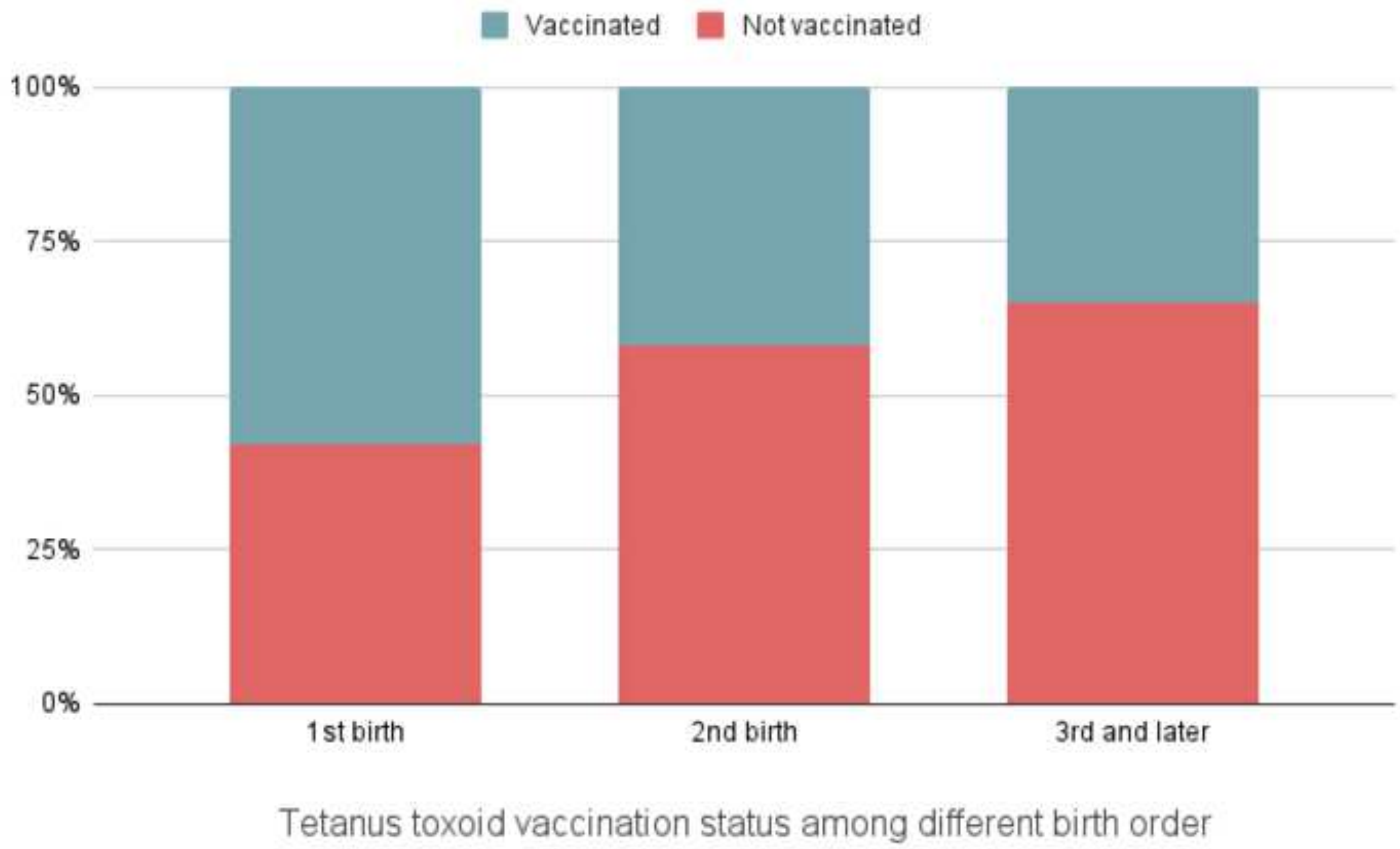

Figure 4

Click here to access/download;Figure;Fig 4.tiff

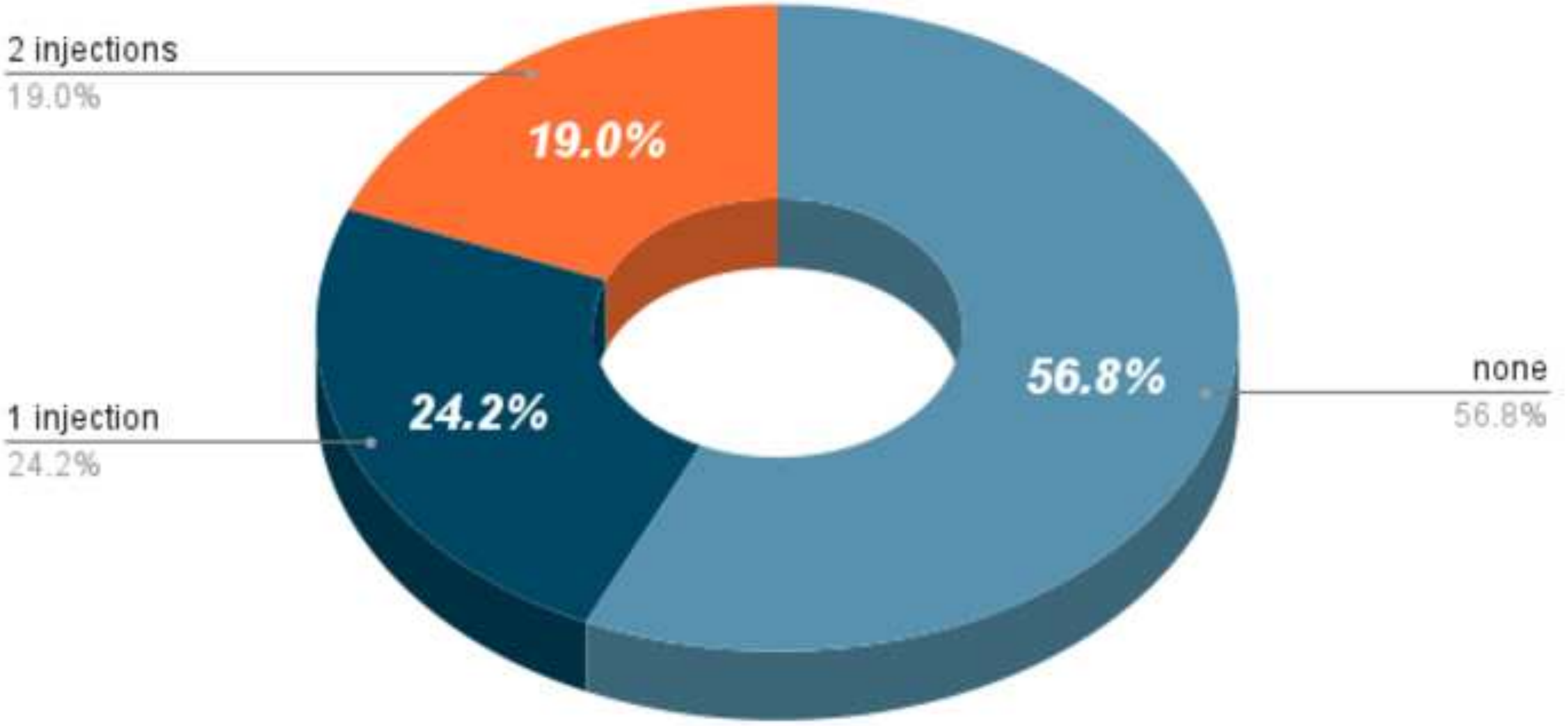

Tetanus Toxoid Vaccination Among Mothers with Neonatal Mortality

Click here to access/download
Supporting Information
Supplementary Appendix.docx

A Study of Association Between Maternal Tetanus Toxoid Immunization and Neonatal Mortality in the Context of Bangladesh

Sujan Kumar Naha[1*], Md. Efty Islam Arpon[1*], Rifa Tasfia Siddique[1], Farjana Rahman Ripa[1], Mohammad Nayeem Hasan[1], Md Jamal Uddin[1,2**]

[1]Department of Statistics, Shahjalal University of Science & Technology, Sylhet-3114, Bangladesh.

[2]Department of Graduate Studies, Daffodil International University, Dhaka, Bangladesh

*Equal contributions

****Corresponding Author: Md Jamal Uddin; jamal-sta@sust.edu; Department of Statistics, Shahjalal University of Science and Technology, Sylhet-3114, Bangladesh.**

Abstract

Background: Maternal tetanus toxoid (MTT) vaccination during pregnancy remains an important factor for reducing infant mortality globally, especially in developing nations, including Bangladesh. Despite commendable progress in reducing child mortality through widespread MTT vaccination during pregnancy, the issue still exists. This analysis explores the impact of MTT vaccination on neonatal mortality in Bangladesh and identifies associated factors.

Methods: This research utilizes data from the 2019 Bangladesh Multiple Indicator Cluster Survey (MICS). The dataset consists of 23,402 cases;~~:,~~ among them, 587 cases resulted in ~~the~~ infant death. The outcome variable was infant mortality, which was binary. ~~and~~ The independent variables identified as potential contributors to the cause of death included tetanus toxoid vaccination status, mode of delivery (cesarean section or not), and mother's education level, among others. The Poisson model was employed to analyze the ~~analyses~~ data.

Results: The analyses showed that the neonatal mortality rate was 2.51%. Notably, 45.90% of mothers received the MTT vaccination during pregnancy. Among them, 23.07% received a single dose, and 22.82% took adequate doses (receiving more than two doses) and adhered to WHO guidelines. The

Formatted: Left, Line spacing: Double

adjusted incidence rate ratio (IRR) was 1.36, which indicates that there was a 36% higher risk of neonatal mortality for those children whose mothers did not take TT (IRR = 1.36, p = 0.081). We also found that women from middle-class households (IRR = 1.58, 95% CI = 0.98, 2.54) and women with higher parity (IRR = 1.96, 95% CI = 0.95, 4.03) also had a higher risk of newborn fatalities. A comparable trend has been observed regarding the correlation between the number of tetanus doses administered and neonatal mortality, where it also emphasizes the importance of receiving adequate doses (a minimum of 2 doses of tetanus vaccine) to mitigate neonatal mortality (adjusted IRR = 0.54, 95% CI = 0.29, 1.01) in comparison to no doses received. ~~The crude incidence rate ratio (IRR) was 1.19,~~ which ~~indicates that there was~~ a ~~19% higher risk of neonatal mortality for those children whose mother did not take TT (p = 0.181), and the adjusted IRR was 1.36,~~ which ~~indicates~~ a ~~36% higher risk of neonatal mortality (adjusted IRR = 1.36, p = 0.081).~~

Formatted: English (United States)

Conclusion: ~~Maternal Tetanus Toxoid vaccination plays a crucial role in reducing child mortality.~~Administering a minimum of one maternal tetanus dose significantly lowers the risk of neonatal mortality. Other than Maternal Tetanus Toxoid vaccination, the analyses underscore~~s~~ various contributors to neonatal mortality, encompassing maternal healthcare~~, vaccination~~, delivery procedures, socio-economic status, and education. Targeted interventions addressing these factors have the potential to efficiently decrease neonatal mortality rates and improve overall maternal and child health.

Formatted: Font: Not Bold

Keywords: Tetanus, tetanus toxoid vaccine, neonatal death, neonatal mortality, maternal tetanus, adequate doses.

Introduction

Tetanus is an acute infectious disease that continues to pose a significant public health challenge. ~~Neonatal tetanus is a life-threatening disease that can be prevented.~~ It occurs when the umbilical stump is covered with contaminated material or when non-sterile tools are used to cut the umbilical cord ~~Neonatal tetanus is a life-threatening but preventable disease that develops when contaminated material is used to cover the umbilical stump or when non-sterile tools are used to cut the umbilical cord~~ (1). ~~This common bacteria is typically found in soil, but it can also be found in human and domestic animal feces This common bacteria lives in soil by nature, however, it can also be isolated from human and domestic animal feces .~~ It can complicate surgery, intramuscular injections, gangrene, burns, ulcers, necrotic snakebites, middle ear infections, septic abortions, and childbirth ~~It can make surgery, intramuscular injections, gangrene, burns, ulcers, necrotic snakebites, middle ear infections, septic abortions, and childbirth more difficult~~ (2). Tetanus can impact individuals across all age groups; however, it presents a particularly significant risk to newborns and pregnant women lacking adequate tetanus-toxoid vaccinations. In such cases, the disease can often result in death or significant health complications~~While tetanus can impact anyone, it is particularly severe in newborns and pregnant women who lack adequate tetanus-toxoid vaccinations, often leading to death or significant health issues~~ (3).

Formatted: Left

The disease is caused by *Clostridium tetani* bacteria. This common bacterium is typically found in soil, but it can also be found in human and domestic animal feces (4). Spores of *Clostridium* ~~t~~*Tetani* enter the human body through wounds or small abrasions when anaerobic conditions are met. Tetanus can be avoided ~~whenever a wound arises~~ with proper ~~wound~~ care whenever a wound arises and adequate vaccination (5). However, it should be noted that tetanus cannot be completely eliminated due to the presence of spores in the natural environment.

Formatted: Not Highlight

Maternal and neonatal tetanus (MNT) is a significant public health issue that impacts mothers and their newborns. It is prevalent when women give birth in unsanitary conditions and do not receive the complete series of tetanus toxoid (TT) vaccinations. So, Nonetheless, anone effective approach to reducing the spread of the disease is through the immunization of newborns and the women who are planning to become pregnant. This immunization can be achieved by vaccination with tetanus-toxoid-containing vaccines (TTCV). Maternal and neonatal tetanus (MNT) is a significant public health issue that impacts mothers and their newborns. It occurs when women give birth in unsanitary conditions and do not receive the complete series of tetanus toxoid (TT) vaccinations. Maternal and neonatal tetanus (MNT) protection through vaccination ensures that a baby remains protected from the disease during the initial two months of life However, tetanus cannot be eliminated since the spores occur naturally in the environment, but by immunizing newborns and women who will become pregnant, the chance of spreading the disease can be reduced. A major public health problem called maternal and neonatal tetanus (MNT) affects mothers and their newborns when they give birth in unhygienic surroundings and do not receive the full course of tetanus toxoid (TT) vaccinations. A baby is shielded from the disease for the first two months of life if the mother has received maternal and neonatal tetanus (MNT) protection (6).

Tetanus can be prevented by vaccination withThe tetanus-toxoid-containing vaccines (TTCV) , which are typically given to reproductive women between the ages of 15 and 44 years to protect both mother and newborn and prevent maternal and neonatal tetanus. A woman needs five tetanus toxoid doses to be protected against tetanus for life (6). For children, the World Health Organization (WHO) recommends six doses of TTCV, three of which are primary and three of which are boosters. The three-dose primary series should start as early as six weeks of age, with the following doses spaced at least four weeks apart. Preferably, the three booster doses should be administered between the ages of 4 and 7 years, 12 and 23 months, and 9

Formatted: Left

Formatted: Not Highlight

Formatted: Not Highlight

Formatted: Not Highlight

Formatted: Not Highlight

Formatted: Not Highlight

Formatted: Font: (Default) Times New Roman, 12 pt, Font color: Black, English (United States)

and 15 years (7). Booster immunization campaigns are directed toward women of childbearing age, and in many nations, they have significantly decreased the incidence of tetanus in both mothers and newborns (5).

It has been challenging to enhance neonatal health in low-income countries because babies have less access to health care and are thus more at risk of worse health outcomes (8).~~;~~ F~~f~~or instance, a prior study between 1997 and 2002 discovered that 54.7% of newborn deaths happened during the neonatal period ~~between 1997 and 2002~~ (9). ~~-~~A failure in routine prenatal immunization programs, below average antenatal care services, an increase in home deliveries, and unhygienic deliveries result in a mortality rate of about 35% and proximal (309000) deaths from maternal or neonatal tetanus (10). Tetanus caused an estimated 787,000 infant fatalities in 1988, with an estimated proportionate mortality rate of 6.7 per thousand live births, demonstrating the disease's significant contribution to the world's high rate of neonatal mortality (11). Globally, reported newborn tetanus mortality dropped by 85% between 2000 and 2018, from 170,829 to 25,000 deaths (12). ~~-~~Approximately 25,000 fewer newborns died in 2018 compared to 215,000 in 1999, a considerable decline since that year. Out of 59 low- and middle-income at-risk nations, MNT has currently been eradicated in 47 of them (13).

The Bangladesh Demographic and Health Survey reported neonatal mortality rates of 28 per 1,000 live births in 2014 and 30 in 2017, while under-five mortality rates were 46 per 1,000 in 2014 and 45 in 2017 (16,17). The current rate is significantly higher than the global average of 6.6 per 1,000 live births in 2015 and exceeds the 7.4 per 1,000 averages in developing countries and neighboring countries like India and Nepal (18). The WHO reports that preterm birth causes 30% of global neonatal deaths, sepsis or pneumonia 27%, birth asphyxia 23%, congenital abnormalities 6%, neonatal tetanus 4%, diarrhea 3%, and other causes 7% (19–21). An exploratory study in rural Bangladesh found that tetanus accounts for 42% of newborn deaths (22). According to another study, out of 330 infant deaths, 112 of them fit the tetanus case description (23). A study in 2016 found that t~~T~~here were 24.4 neonatal deaths for every 1000 live births in Bangladesh,

Formatted: Indent: First line: 0.5"

based on 6748 neonatal fatalities spread throughout four districts (Thakurgaon, Jamalpur, Moulvibazar, and Narail), with a combined population of 6.7 million (24). ~~Despite TT2's estimated 45% efficacy, maternal history of receiving two doses of the tetanus toxoid did not lower the risk in Bangladesh .~~

Tetanus is still prevalent and a major cause of death in low-income countries, despite considerable World Health Organization programs that effectively target maternal and newborn disease (14). As opposed to low-income countries (LICs), High-Income Countries (HICs) like the United States of America, England, and Denmark have long since managed tetanus-related newborn mortality (15). A few Low and Middle-Income Countries (LMICs), like Bangladesh, Afghanistan, and Nepal, are also making progress in lowering neonatal tetanus fatality rates (11) even though the pace of reduction is very low (16).

Effective intervention programs have been implemented in these countries, which include improved cord care, increased tetanus toxoid coverage for pregnant women, adoption of safe birth practices, and implementation of postpartum care procedures. Despite some successes in countries such as Bangladesh and Nepal, low- and middle-income countries (LMICs) still face challenges in reducing infant mortality caused by neonatal tetanus. ~~Effective intervention programs have been put in place in these nations, including better cord care, better tetanus toxoid coverage for expectant women, safe birth practices, and postpartum care procedures. Although there have been some successes in nations like Bangladesh and Nepal, LMICs continue to struggle with the reduction of infant mortality for neonatal tetanus.~~ According to our knowledge, no study ever before was able to give a clear picture of the influence of maternal tetanus toxoid vaccination in reducing neonatal mortality in Bangladesh. The objective of our study was to investigate the potential link between neonatal mortality and tetanus toxoid immunization among women aged 15-49 in Bangladesh. This study will assist policymakers and planners in making more informed and effective decisions regarding this important issue.~~We aimed to examine the association between neonatal mortality and tetanus toxoid~~

Formatted: Justified, Indent: First line: 0.5", Line spacing: Double

immunization among women aged between 15-49 in the context of Bangladesh. This study will help the policymakers and planners to take more convenient and efficient steps regarding this vital issue.

Formatted: English (United States)

Formatted: Line spacing: single

Materials and methods

Description of the dataset:

Formatted: Not Highlight

The research was conducted using a cross-sectional study design, utilizing the accessible data from the Bangladesh Multiple Indicator Cluster Survey (MICS) conducted by the Bangladesh Bureau of Statistics (BBS) in collaboration with the United Nations International Children's Emergency Fund (UNICEF) in 2019. The survey was carried out from January 19 to June 1, 2019. The survey sample was selected through a two-stage, stratified cluster sampling method in order to recruit the participants. The 2011 Bangladesh Census of Population and Housing was used as the sampling frame for this study. The enumeration areas (EAs) selected for the census enumeration were the primary sampling units (PSUs) selected during the initial stage. In each sample, a household listing was completed as part of the research process. In the second stage, a sample of households was selected for further analysis. The sample was created to generate estimates for various variables concerning women and children across the entire country, including both urban and rural areas in all 64 districts of the seven administrative divisions. The survey consisted of a sample of 64,400 households and approximately 3,220 primary sampling units (PSUs) This research was performed based on a cross-sectional study of the 2019 accessible data available from the Bangladesh Multiple Indicator Cluster Survey (MICS), which was carried out in 2019 from January 19 to June 1 by the Bangladesh Bureau of Statistics (BBS) with collaboration from United Nations International Children's Emergency Fund (UNICEF). The survey sample was chosen using a two-stage, stratified cluster sampling procedure to recruit the participants. The 2011 Bangladesh Census of Population and Housing served as the base for the sampling frame. The

Formatted: Not Highlight

enumeration areas (EAs) chosen for the census enumeration were the primary sampling units (PSUs) chosen at the initial stage. In each sample EA, a household listing was completed, and in the second stage, a sample of households was chosen. The sample was created to give estimates of a broad variety of variables for women and children nationwide, including those for urban and rural locations throughout all 64 districts of the seven administrative divisions. The survey included a sample of 64,400 households and about 3,220 primary sampling units (PSUs) (25). The households were properlythoroughly interviewed by trained interviewers using questionnaires that covered reproductive history, prenatal and postnatal care, family planning, and demographic characteristics. The datasets were made available to the public for access and were specifically collected from sampling units (PSUs) Trained interviewers used questionnaires covering reproductive history, prenatal and postnatal care, family planning, and demographic characteristics to properly interview these households. Datasets were open access for the public domain (26).

Children who have passed away during the neonatal period or within the first month after birth may be considered as eligible cases for participation in the study. Moreover, it is important to note that the study subjects were specifically chosen to include children who had a high risk of newborn mortality, even though they were still alive at the time of the survey. Births that occurred prior to 2014 or five years before the survey are not classified as cases.

Formatted: Indent: First line: 0.5", Line spacing: Double

Children who have died in the neonatal period or in the first month after birth may be considered eligible cases for participation. Furthermore, although they were still alive at the time of the survey, children who had a high risk of newborn mortality were specifically selected as study subjects. Births that took place before 2014 or five years before the survey are not considered cases.

Study Variables

The outcome variable is the child's survival status in the neonatal stage, which is binary and classified as either 0 (for alive) or 1 (for death). The analysis utilized data on the age at which mortality events occurred among live births that took place in the five-year period prior to the MICS 2019 survey. The variable indicating whether or not a neonatal death occurred was recoded using the standard procedures outlined in the SPSS syntax files provided by UNICEF outlined in the SPSS syntax files for the 2019 survey as provided by UNICEFThe child's survival status in the neonatal stage, which is binary and classified as either 0 (for alive) or 1 (for death), is the outcome variable. The analysis employed data pertaining to the age at which mortality events occurred among live births that transpired during the five-year period immediately preceding the MICS 2019 survey. The variable identifying if the neonatal death occurred or not was recoded using the standard procedures following the SPSS syntax files for the 2019 survey provided by UNICEF (27). TheThese recoded data were used to create the outcome variable for the research analysis.These data were utilized in the construction of the outcome variable for the research analysis.

The principal factor under investigation as a potential exposure variable was the administration of the tetanus toxoid vaccine, classified as either "0" for non-taken or "1" for taking at least 1 dose. Again, at least two doses are considered adequate according to the WHO. In addition to the principle factor, the additional modifiable risk factors analyzed in this research consisted of cesarean section (yes, no), gender of the neonate (boy and girl), antenatal care visits (less than 4, 4 and above), division (Barisal, Chittagong, Dhaka, Khulna, Rajshahi, Rangpur, and Sylhet), mother's education level (primary, higher secondary), birth order (1, 2, and 3), mother's age during pregnancy (15-20, 20-24, 25-29, 30-34, 35-39, 40-44, and 45-49), socioeconomic status (poorest, middle, and richest).

Statistical Analysis

Formatted: Indent: First line: 0.5", Line spacing: Double

Formatted: Not Highlight

Formatted: Not Highlight

Formatted: Left, Indent: First line: 0", Line spacing: single

Formatted: Indent: First line: 0.5"

Chi-square tests have been implemented to assess the relationship between potential exposure or control variables and infant mortality, forming the foundation for our models. Factors in bivariate models with p-value < 0.2 were included in the multivariable model, along with a previous multicollinearity test, and this threshold for p-value has been taken from a previously published paper (28,29).

For highly skewed binary data with significant class imbalance, Poisson regression can be preferable over logistic regression. Poisson regression performs better because of its log transformation, which improves results stability and interpretability. ~~Poisson regression provides better performance due to its log transformation, which enhances stability and interpretability of results.~~ This approach is particularly advantageous in cases of high skewness, as it offers reliable estimates and reduces the risk of overfitting. The Poisson model effectively estimates relative risk and manages variance overestimation, making it a robust choice for binary outcomes in such contexts (30,31). Our data's extreme imbalance and skewness align well with Poisson regression's strengths. Therefore, the final analysis was carried out using a Poisson regression model to examine the relationship between the outcome variable and several predictor variables, since in our data the proportion of death is less than 5% (approximately 2.54%), indicating a high skewness in child mortality. The model included all variables deemed significant by chi-square analysis, ensuring a comprehensive examination of their impact on child mortality. The missing values were removed with case-wise deletion as per the default data handling procedure in STATA. For each predictor variable, the incidence rate ratio (IRR)~~,~~ and the 95% confidence interval (CI) for child deaths were calculated.

Ethics Statement

The analysis utilized publicly accessible datasets from health surveys, ensuring that all identifiable personal information was removed. The analysis conducted in this study utilized secondary data sources, thereby exempting it from the need for ethical review approval from the relevant institutions. Nonetheless,

Formatted: Left

the methods employed in MICS underwent a thorough review and received approval from both UNICEF and BBS.~~Our analysis was based on publicly available datasets of health surveys and removed all the personal information that could be identifiable. As the study analysis was made from a secondary source of data, it was exempt from ethical review approval from the respective institutions. However, MICS methods were reviewed and approved by both UNICEF and BBS.~~

Results

The most recent MICS (2019) survey data were integrated into this study. Among the 23,402 neonatal births, there were 587 neonatal fatalities, accounting for approximately 2.54% of the total neonatal births (Fig. 1). Approximately 45.9% of women received at least one dose of tetanus toxoid immunization during their most recent pregnancy, while 22.8% of women received the adequate number of doses. A multitude of factors played a role in the occurrence of early newborn deaths. In the univariate analysis presented in Table 1, it was observed that 67.2% of women underwent cesarean section, while the percentage of normal deliveries stood at 32.8%. Notably, the death rate associated with cesarean sections was low, recorded at 1.7%. Observations indicate that neonatal mortality rates are elevated in boys at 2.7%, compared to 2.3% in girls during delivery. It is worth mentioning that the neonatal mortality rates were 1.7% for mothers who had fewer than four antenatal care (ANC) visits. On the other hand, mothers who had four or more ANC visits had a slightly lower neonatal mortality rate of 1.21%. Furthermore, a minor percentage of mothers (0.13%) reported uncertainty regarding the number of antenatal care visits they had attended. Notably, a substantial correlation in univariate analysis has been identified between division and neonatal mortality, with mothers from the Sylhet division experiencing the highest neonatal mortality rate at 3.7%. It is important to highlight that education significantly influences neonatal mortality rates. Women with primary or secondary education experienced a 2.7% rate of neonate deaths, while those with higher secondary education had a rate of 1.6%. Birth order appears to be an important factor, as second-parity births are linked to increased neonatal mortality, highlighting the need for further investigation into the underlying causes. Women with increased birth parity experience elevated neonatal mortality, which constitutes another critical factor. The wealth index of the respondents indicates that 45.6% came from poor families, 37.2% were from the middle class, and 16.7% belonged to the rich category. The neonatal mortality rates are observed to be 2.9% for the poorest, 2.4% for the middle class, and 1.7% for the richest segments of the population. Finally, it is noteworthy that

Formatted: Indent: First line: 0"

a considerable proportion of women aged 20–24 experienced a significant rate of newborn infant mortality, recorded at 2.9%.Data from the most recent MICS (2019) survey were incorporated into this research. Of the 23,402 neonatal births, 587 resulted in neonatal fatalities or, roughly 2.54% of all neonatal births (Fig 1). About 45.9% of women took at least one dose of tetanus toxoid immunization during their last pregnancy and 22.8% of women took adequate doses. Numerous variables contributed to early newborn deaths. For example, in the univariate analysis provided in Table 1, 67.2% women were delivered by cesarean section whereas the percentage of normal delivery was 32.8% (deathrate was low in cesarean section by 1.7%). It was also observed that neonatal mortality is higher in boys (2.7%) than in girls (2.3%) during delivery. Notably, neonatal mortality rates were 1.7% among mothers who had fewer than four ANC visits, whereas mothers who had four or more ANC visits, experienced a slightly lower neonatal mortality rate of 1.21%. Additionally, a small portion of mothers (0.13%) indicated that they did not know the number of antenatal-care they have taken. Interestingly, a significant relationship in univariate analysis has been found between division and neonatal mortality where mothers of Sylhet division had the highest neonatal mortality (3.7%). An additional point that can be noted is that education plays a significant role in neonatal mortality. Women who took primary or secondary education faced 2.7% neonate death and higher secondary level had 1.6%. Birth order also emerges as significant, with second-parity births associated with higher neonatal mortality, calling for a deeper exploration of underlying factors. Women with higher birth parity deal with higher neonatal mortality which is another significant factor. In terms of the wealth index of the respondents, 45.6% were from poor families, 37.2% were from the middle class, and 16.7% were from the rich. Among them the poorest deals with 2.9%, the middle 2.4%, and the richest 1.7% of neonatal mortality respectively. Last but not least, the majority of women in the 20–24 age range had a significant rate of newborn infant mortality (2.9%).

Fig 1. Proportion of dead and alive neonates

Table 1 displays the mortality status row proportions across ~~various~~different covariate categories. It is evident from the table that the respondents classified in the lowest wealth index category experience the highest percentage of neonatal deaths at 2.9%, in contrast to the richest group at 1.7% and the middle class at 2.4%. The Sylhet division exhibits the highest proportion of neonatal mortality at 3.7%, closely followed by the Rajshahi division at 2.9%. The mortality rate is notably elevated in individuals with a third party or higher, recorded at 3.1%. Additionally, mothers who experienced natural births exhibit a greater percentage of neonatal deaths (4.8%) in contrast to those who underwent cesarean sections (3.1%). Finally, the mothers who received the tetanus toxoid vaccine exhibited a lower neonatal mortality rate of 3.3% compared to the 2.9% observed in non-vaccinated mothers during their last pregnancy.

~~From Table 1, we get the row proportions of the mortality status in different categories of the covariates. Notably, from the table we see that the respondents in the poorest category of the wealth index have the highest proportion of neonatal deaths (2.9%) compared to the richest (1.7%) and middle class (2.4%). The proportion of neonatal mortality is also highest in the Sylhet division (3.7%) followed by the Rajshahi division (2.9%). The proportion of death is also highest in the 3rd parity or more (3.1%). Furthermore, the mothers who had natural birth have a higher proportion of neonatal deaths (4.8%) compared to mothers who had births by cesarean section (3.1%). Lastly, the vaccinated mothers with the tetanus toxoid vaccine had lower neonatal mortality (3.3%) as compared to non-vaccinated mothers during their last pregnancy (2.9%).~~

[Table 1 Here]

From the figures, we get an insight into the association and distribution among some variates. The second figure (Fig. 2) highlights the importance of tetanus vaccination in reducing neonatal deaths. Regions with higher vaccination rates among neonates, such as Barishal, show lower percentages of neonatal deaths. Conversely, areas like Sylhet, which have lower immunization rates, have a greater incidence of infant fatalities. This pattern underscores the critical role of tetanus vaccination in improving neonatal survival rates, as evidenced by the significantly lower mortality rates in areas with better vaccination coverage. (Fig. 3) suggests that first-–parity births have the highest rate of vaccination, and with subsequent births, the proportion of vaccinated individuals decreases. Relating this to Table 1, it is observed that the 3rd or higher parity births also have a higher proportion of neonatal deaths, implying the importance of vaccination. (Fig. 4) represents the proportion of doses of the tetanus toxoid vaccine taken by the mother, where the newborn faced a neonatal death. —Further implying the significance of adequate tetanus toxoid vaccination, it is observed that, among the neonatal deaths, a significantly large proportion of mothers (56.8%) had taken no tetanus toxoid vaccine during pregnancy, and only 19% of the mothers had an adequate amount of at least 2 doses of tetanus toxoid vaccine.

Fig 2. Tetanus toxoid vaccination status across different divisions

Fig 3. Tetanus toxoid vaccination status among different birth order

Fig 4. Tetanus Toxoid Vaccination Among Mothers with Neonatal Mortality

The results of the regression analysis of the factors that predict newborn mortality are shown in Table 2. In the Poisson regression model, including the variables with $p < 0.2$, $p < 0.1$, $p < 0.05$, $p < 0.01$, $p < 0.001$ $p < 0.2$, $p < 0.1$, $p < 0.05$, $p < 0.01$, $p < 0.001$ iin the univariate analysis, tetanus doses, women's education level, women with a higher index wealth quintile, and women with a higher parity were found to be significantly

linked with newborn mortality in the Poisson regression model. Crude incidence rate ratios (IRRs) for death are shown in the characteristics table for women who had tetanus toxoid (TT) immunization vs. those who did not. When compared to the vaccinated group, the unvaccinated group's crude IRR for death was 1.19 at first, with a p-value of 0.18, indicating no discernible difference in mortality. However, after adjusting for the other factors in the model, the crude IRR rose to 1.36 with a p-value of 0.08. Although this is not statistically significant at the traditional cutoff point of $p < 0.05$, this adjusted IRR suggests an elevated risk of MTT on infant mortality. According to our analysis, mothers with no tetanus dose had a 36% increased incidence rate ratio of experiencing newborn mortality (IRR = 1.36, 95%CI = 0.96, 1.93) compared to mothers taking at least a single tetanus dose. A similar trend has been found in the relationship between the number of tetanus doses taken and neonatal mortality that has been given in Appendix 1, which also describes taking adequate doses (at least 2 doses of tetanus vaccine) to reduce neonatal mortality (Crude IRR = 0.87, 95%CI = 0.57, 1.32) compared to none taken host. Interestingly, the cesarean section was found insignificant. We also discovered that women's education level was significantly associated with newborn mortality, where mothers with primary or secondary education had an increased 86% incidence rate ratio of newborn mortality (IRR = 1.86, 95%CI = 1.16, 2.99) compared to mothers who had studied at the higher secondary level. Additionally, women from poor families had a significantly higher incident rate ratio compared to the women from rich families (IRR = 1.77, 95%CI = 0.93, 3.38). Similarly, women from middle-class families had a significantly higher incident rate ratio compared to the women from rich families (IRR = 1.58, 95%CI = 0.98, 2.54). In addition, third-–parity births wereas found to be substantially correlated with newborn mortality when compared to the second--parity births (IRR = 1.96, 95%CI = 0.95, 4.03). This indicates that women with higher parity had a 96% increased risk of neonatal deaths. Women aged between 25 andto 29 years indicated a fairly significant association (IRR = 1.89, p = 0.081) with newborn death, showing an 89% higher incident rate ratio of neonatal mortality compared to women aged between 30 andto 35 years.

Formatted: Font color: Text 1

Likewise, women aged between 45 andto 49 years had a significantly (IRR = 4.05, p = 0.055) higher rate ratio thanagainst women aged between 30 andto 35 years. Gender, antenatal care visits, and division, which are the necessary factors, were found to be insignificant in the model. Similar trend has been found in the relationship between number of tetanus doses taken and neonatal mortality that has been given as appendix 1, which also describes taking adequate doses (at least 2 doses of tetanus vaccine) reduce neonatal mortality (Crude IRR = 0.87, 95%CI = 0.57, 1.32) comparing to none taken host.

[Table 2 here]

Discussion

Tetanus demonstrates a significant threat to human life Tetanus poses a serious risk to life (32). Despite significant advancements over the past twenty years in reducing global tetanus-related fatalities, neonatal tetanus has garnered considerable focus, while maternal tetanus remains largely underexplored Though remarkable progress has been made for the last two decades in lowering tetanus-related deaths globally, neonatal tetanus has received a lot of attention up to this point and very little is known to maternal tetanus (15,33). This survey aims to identify the impact of neonatal mortality in relation to tetanus toxoid vaccination among pregnant women aged 15 to 49 years. The findings indicate that maternal vaccination plays a significant role in decreasing neonatal death rates.For this very reason, this survey is conducted to identify the result of neonatal mortality by taking tetanus toxoid vaccination among pregnant women aged between 15-49 years and found that maternal vaccination is conspicuous factor in reducing neonatal death.

Formatted: Left

Previous investigations have not explored the relationship between neonatal mortality and tetanus toxoid immunization, in conjunction with other essential covariates. Our primary results demonstrate that women who obtain at least one dose of the tetanus vaccine substantially reduce the likelihood of neonatal

Formatted: Indent: First line: 0.5"

death in their children. Furthermore, around 2.54% of neonatal deaths in Bangladesh are associated with pregnant women who did not receive at least one dose of the tetanus toxoid vaccination. In contrast, about 45.9% of women have received at least one dose of the vaccine, which could serve as valuable data for initiatives aimed at enhancing vaccination rates.

No studies have been conducted before to show the association between neonatal mortality and tetanus toxoid immunization along with other necessary covariates. Our main findings indicate that women who receive at least one dose of the tetanus vaccine significantly lower their child's risk of neonatal death. Additionally, approximately 2.54% of neonatal deaths in Bangladesh can be linked to pregnant women who did not receive at least one dose of the tetanus toxoid vaccination, and approximately 45.9% of women received at least one dose of the vaccine, which may be useful information for efforts to increase vaccination rates.

Our findings show that only 22.82% of pregnant women had received a sufficient amount of the TT vaccine, which is low as compared to a study showing that sufficient TT immunization among pregnant women was 75% worldwide (34). These findings are consistent with previous research's findings, which showed a lower prevalence of obtaining the adequate TT vaccine (23) and the rate of newborn death was 63.8 per 1000 live births (35). The prevalence of TT immunization was 81.8% for MICS (2006) and 61.3% for MICS (2012–2013) (36) and we observed in our research that for MICS (2019), the rate of taking tetanus doses was 54.1%. These findings shed light on the varying levels of vaccination coverage among pregnant women, emphasizing the need to promote and ensure full compliance with the WHO's recommended vaccination regimen during pregnancy for the optimal prevention of maternal and neonatal tetanus. Overall, in Bangladesh, the prevalence has generally been declining decreasing over the course of the survey years.

In our model, we discovered that tetanus doses taken, women's education level, wealth index, and birth order were significantly associated with higher odds of neonatal mortality. Our analysis shows that a

Formatted: Not Highlight

Formatted: Not Highlight

Formatted: Not Highlight

mother with no tetanus doses has the 36.4% more chance to experience neonatal death (IRR = 1.36, 95% CI = 0.96, 1.93) comparededing to a mother with at least 1 dose. Similar to other studies, we discovered that the TT vaccine had a protective effect against infant mortality when compared to IFA (iron–folic – acid) supplementation alone (37–39) The evidence from India aligns with our analysis, indicating that receiving at least one dose of the TT vaccination could decrease infant death (40). Another study revealed a sharp reductiondecrease in newborn mortality as a result of a decrease in neonatal tetanus deaths (41). Tetanus vaccination coverage plays a vital role in reducing neonatal tetanus. We are the first As far as we are concerned, we are the first to show the association between tetanus toxoid vaccination and neonatal mortality in Bangladesh in recent years.

Cesarean section is a potential factor for neonatal mortality according to previous studies, where cesarean section delivery is highly correlated with neonatal mortality in low-income countries like Bangladesh (8,42). However, we find found no association of between neonatal mortality with and cesarean sections, which may be a noticeable gap in our study that could be addressed with the confirmation of further research.

A mother's education significantly plays a vital role in neonatal mortality. Women who only completed elementary or secondary school had a greater risk of newborn mortality compared to women who pursued higher education. Considering that educated women may be better empowered to make decisions about their health, education may increase their understanding of the harmful impacts of tetanus and neonatal death (36,43).

Another important factor associated with neonatal mortality is higher parity. Our study revealed that women with higher parity have lower odds of neonatal mortality compared to those with lower parity. This is supported by findings from previous studies that have found higher parity to be a significant predictor of neonatal mortality (44–46). The past birth experiences of the ladies may be the cause. It's also reasonable

Formatted: Not Highlight

Formatted: Not Highlight

Formatted: Not Highlight

Formatted: Not Highlight

Formatted: Not Highlight

that women with more children are less likely to be employed or to have greater levels of education. There are further studies that show older women in the nation are less likely than younger women to use prenatal and delivery care, which can be another identifiable reason (47–49).

Various age groups in the reproductive age of women play a crucial role in reducing neonatal death. Older women (age 45-49 with IRR = 4.05, 95%~~-~~CI = 0.97, 16.95) are more exposed to neonatal death than any other reproductive period,~~s~~ and it is one of the important issues that needs to be taken care of. Similar studies show that maternal periods in different age groups directly influence neonatal mortality, which is likely due to complications of delivery that arise~~s~~ at a later age (50,51).

The health of the Bangladeshi population has significantly improved over the past 20 years, and Bangladesh has been acknowledged as an example of "good health at low cost" (52). As far as we are aware, socioeconomic status has a big impact and is highly correlated; babies from the "poorest" homes are more likely to die, which emphasizes how critical it is to solve socioeconomic gaps in access to healthcare. Our research says women with wealthy households are less likely to face neonatal mortality compared to women with a poor wealth quantile index. This result is in line with earlier research demonstrating that increasing the wealth index of women living at home protects against tetanus in comparison to a low wealth index (40,43,53,54) We believe that women from wealthy families are more likely to have access to healthcare services than women from low-income families. A policy and programming requirement to ensure that all pregnant women receive at least one dose of prenatal medication, especially those in need or marginalized population subgroups, is expected to have a significant influence on improving neonatal survival. This specific intervention should be within the grasp of the public health system as an immediate priority due to difficulties in providing the entire health system functionality needed for comprehensive and safe mother and newborn care (36).

Formatted: Not Highlight

Formatted: Not Highlight

Formatted: Not Highlight

Formatted: Not Highlight

Formatted: Not Highlight

Formatted: Font color: Auto

Formatted: Not Highlight

Limitations and Strengths

Formatted: Not Highlight

Strengths

To our knowledge, this study is the first in Bangladesh to investigate the relationship between maternal tetanus toxoid vaccination and neonatal mortality. We employed appropriate data analysis techniques, taking into consideration all intricate survey designs. Findings from this work can be used to inform future research, policy, and clinical practice and to benchmark progress. One of the benefits of this study is the sizeable and nationally representative sample size, and the results are applicable to the entire country. The information was gathered using the most recent survey. Our results would surely pique interest in additional research and educate decision-makers about the gaps in tetanus care that need to be filled.

Limitations

However, despite the several strengths, the existence of bias resulting from different survey time points and the cross-sectional character of the data cannot be confirmed. Some variables that were statistically insignificant but still important for the research study have been considered in the model. It is crucial to keep in mind that the mortality that is being discussed here includes deaths from all causes. Regretfully, our analysis lacks particular data that isolates the risk exclusively for newborn mortality. Notwithstanding this restriction, the trend that has been seen points to a higher risk of death for those who have not had the TT vaccination; however, statistical significance is not attained. This realization emphasizes how crucial it is to conduct additional studies to determine the specific effect of the TT vaccine on mortality outcomes, especially neonatal death. To offer more precise information, future research focusing on infant mortality rates and any confounding factors is required.

The level of significance was deemed to be fairly high. In addition, the study's drawback also emerges from the fact that we had little control over the correlated variables to include in the analysis due to the

secondary data source we employed. Conclusions regarding a causal association, the relative contributions of immunization prior versus during the most recent pregnancy, or the best possible ways to increase coverage are not possible due to the cross-sectional character of this investigation. In addition, the dependent variable for newborn death has a large number of missing values, making it impossible to include them in the analysis. This could lead to bias, and the variable varies over time, potentially changing the claimed association in longitudinal studies.

Conclusion

Premature infant death is more common among Bangladeshi women with no tetanus toxoid vaccination. Based on our findingsTo the best of our knowledge gained from our findings, taking at least one maternal tetanus dose greatly reduces neonatal mortality, which clearly shows draw that increasing vaccination coverage as well as deploying the

deploy mandatory law of taking at least one maternal tetanus dose can save infants inat great numbers. Additional investigation is required to determine the pattern of declining infant mortality in Bangladesh following vaccination against maternal tetanus toxoid since there is no evidence related to this. According to our researchFurthermore, Women with higher education levels, those in the wealthiest quintile, and those with greater parity were all significantly linked to reduced odds of newborn mortality women had better education levels, belonged to the quintile with the highest family wealth, and had higher parity were all substantially associated with lower odds of newborn mortality. To decrease neonatal death for the betterment of a country like Bangladesh, we advise taking help from the government and other organizations in conducting immunization campaigns, increasinge vaccination coverage, and improving self-care through proper education and awareness.

Formatted: Indent: First line: 0.5"

485

Declarations

Funding

This research received no specific grant from funding agencies in the public, commercial, or not-for-profit sectors.

Conflicts of interest

The authors reported no potential conflict of interest.

Author Contributions

Conceived and designed the experiments: Md. Efty Islam Arpon, Sujan Kumar Naha. Performed the experiments: Sujan Kumar Naha, Md. Efty Islam Arpon. Analyzed the data: Sujan Kumar Naha, Mohammed Nayeem Hosaain, Md. Efty Islam Arpon. Wrote the paper: Md. Efty Islam Arpon, Sujan Kumar Naha, Rifa Tasfia Siddique, Farjana Rahman Ripa. Supervised by: Dr. Md. Jamal Uddin

The submitted article was authored by all contributors, who also provided their approval.

Acknowledgments

We acknowledge UNICEF and the Bangladesh Bureau of Statistics for allowing us to use the data.

References

1. Edmondson RS, Flowers MW. Intensive care in tetanus: management, complications, and mortality in 100 cases. BMJ [Internet]. 1979 May 26;1(6175):1401–4. Available from: https://www.bmj.com/lookup/doi/10.1136/bmj.1.6175.1401
2. Sievert DM, Rudrik JT, Patel JB, McDonald LC, Wilkins MJ, Hageman JC. Vancomycin-resistant Staphylococcus aureus in the United States, 2002-2006. Clin Infect Dis. 2008 Mar 1;46(5):668–74.
3. Thwaites CL, Beeching NJ, Newton CR. Maternal and neonatal tetanus. The Lancet. 2015 Jan;385(9965):362–70.
4. Wilkins CA, Richter MB, Hobbs WB, Whitcomb M, Bergh N, Carstens J. Occurrence of Clostridium tetani in soil and horses. S Afr Med J. 1988 Jun 18;73(12):718–20.
5. Khan MAS, Hasan MJ, Rashid MU, Kha Sagar S, Khan S, Zaman S, et al. Factors associated with in-hospital mortality of adult tetanus patients-a multicenter study from Bangladesh. PLoS Negl Trop Dis. 2022 Mar;16(3):e0010235.
6. Cook TM, Protheroe RT, Handel JM. Tetanus: a review of the literature. Br J Anaesth. 2001 Sep;87(3):477–87.
7. Tetanus vaccines: WHO position paper – February 2017 [Internet]. [cited 2024 Jun 22]. Available from: https://fctc.who.int/publications/i/item/tetanus-vaccines-who-position-paper-february-2017
8. Goldenberg RL, McClure EM. Maternal, fetal and neonatal mortality: lessons learned from historical changes in high income countries and their potential application to low-income countries. Matern Health Neonatol Perinatol. 2015;1:3.
9. Titaley CR, Dibley MJ, Agho K, Roberts CL, Hall J. Determinants of neonatal mortality in Indonesia. BMC Public Health. 2008 Jul 9;8:232.
10. Gelaw T, Ayalew S, eyene K. KNOWLEDGE AND UPTAKE OF TETANUS TOXOID VACCINE AND ASSOCIATED FACTORS AMONG REPRODUCTIVE AGE GROUP WOMEN IN HAYK TOWN SOUTH WOLLO, ETHIOPIA, CROSS-SECTIONAL STUDY. medRxiv [Internet]. 2022 Dec 21 [cited 2024 Jun 22];2022.12.20.22283731. Available from: https://www.medrxiv.org/content/10.1101/2022.12.20.22283731v1
11. Waiswa P, Kallander K, Peterson S, Tomson G, Pariyo GW. Using the three delays model to understand why newborn babies die in eastern Uganda. Trop Med Int Health. 2010 Aug;15(8):964–72.
12. Njuguna HN, Yusuf N, Raza AA, Ahmed B, Tohme RA. Progress Toward Maternal and Neonatal Tetanus Elimination - Worldwide, 2000-2018. MMWR Morb Mortal Wkly Rep. 2020 May 1;69(17):515–20.
13. Eliminating a silent killer — maternal and neonatal tetanus | UNICEF [Internet]. [cited 2024 Jun 22]. Available from: https://www.unicef.org/blog/eliminating-silent-killer-maternal-neonatal-tetanus
14. Olum S, Eyul J, Lukwiya DO, Scolding N. Tetanus in a rural low-income intensive care unit setting. Brain Commun. 2021;3(1):fcab013.
15. Khan AA, Zahidie A, Rabbani F. Interventions to reduce neonatal mortality from neonatal tetanus in low and middle income countries--a systematic review. BMC Public Health. 2013 Apr 9;13:322.
16. Dhaka A. BANGLADESH DEMOGRAPHIC AND HEALTH SURVEY 2014 National Institute of Population Research and Training Ministry of Health and Family Welfare Dhaka, Bangladesh [Internet]. 2016 [cited 2024 Jul 29]. Available from: www.mitra.bd.com.

17. NIPORT NI of PR and T, Welfare M of H and F, ICF. Bangladesh Demographic and Health Survey 2017-18 [Internet]. 2020 [cited 2024 Aug 7]. Available from: https://dhsprogram.com/publications/publication-FR344-DHS-Final-Reports.cfm
18. McAllister DA, Liu L, Shi T, Chu Y, Reed C, Burrows J, et al. Global, regional, and national estimates of pneumonia morbidity and mortality in children younger than 5 years between 2000 and 2015: a systematic analysis. Lancet Glob Health [Internet]. 2019 Jan 1 [cited 2024 Aug 7];7(1):e47–57. Available from: https://pubmed.ncbi.nlm.nih.gov/30497986/
19. Lawn JE, Cousens S, Zupan J. 4 million neonatal deaths: when? Where? Why? Lancet [Internet]. 2005 Mar 5 [cited 2024 Aug 7];365(9462):891–900. Available from: https://pubmed.ncbi.nlm.nih.gov/15752534/
20. The World Health Report 2005: Make Every Mother and Child Count - World Health Organization - Google Books [Internet]. [cited 2024 Aug 7]. Available from: https://books.google.com.bd/books?hl=en&lr=&id=4Fw0DgAAQBAJ&oi=fnd&pg=PR3&ots=5NqX5YVf-p&sig=b478n48d-bN9rsMaO7A2FfFFddU&redir_esc=y#v=onepage&q&f=false
21. Bryce J, Boschi-Pinto C, Shibuya K, Black RE. WHO estimates of the causes of death in children. Lancet [Internet]. 2005 Mar 26 [cited 2024 Aug 7];365(9465):1147–52. Available from: https://pubmed.ncbi.nlm.nih.gov/15794969/
22. Chowdhury ME, Akhter HH, Chongsuvivatwong V, Geater AF. Neonatal mortality in rural Bangladesh: an exploratory study. J Health Popul Nutr. 2005 Mar;23(1):16–24.
23. Hlady WG, Bennett J V, Samadi AR, Begum J, Hafez A, Tarafdar AI, et al. Neonatal tetanus in rural Bangladesh: risk factors and toxoid efficacy. Am J Public Health. 1992 Oct;82(10):1365–9.
24. Halim A, Dewez JE, Biswas A, Rahman F, White S, Van Den Broek N. When, Where, and Why Are Babies Dying? Neonatal Death Surveillance and Review in Bangladesh. PLoS One [Internet]. 2016 Aug 1 [cited 2024 Jun 22];11(8):e0159388. Available from: https://journals.plos.org/plosone/article?id=10.1371/journal.pone.0159388
25. Bangladesh 2019 MICS Report_English. [cited 2024 Jun 23]; Available from: https://www.unicef.org/bangladesh/media/3281/file/Bangladesh%202019%20MICS%20Report_English.pdf
26. Surveys - UNICEF MICS [Internet]. [cited 2024 Aug 7]. Available from: https://mics.unicef.org/surveys
27. Tools - UNICEF MICS [Internet]. [cited 2024 Aug 6]. Available from: https://mics.unicef.org/tools?round=mics6#analysis
28. Hasan MN, Chowdhury MAB, Jahan J, Jahan S, Ahmed NU, Uddin MJ. Cesarean delivery and early childhood diseases in Bangladesh: An analysis of Demographic and Health Survey (BDHS) and Multiple Indicator Cluster Survey (MICS). PLoS One. 2020 Dec 3;15(12):e0242864.
29. Bursac Z, Gauss CH, Williams DK, Hosmer DW. Purposeful selection of variables in logistic regression. Source Code Biol Med. 2008 Dec 16;3(1):17.
30. Cleophas TJ, Zwinderman AH. Regression Analysis in Medical Research: For Starters and 2nd Levelers. Regression Analysis in Medical Research: For Starters and 2nd Levelers. 2021 Apr 2;1–475.
31. Cleophas TJ, Zwinderman AH. Poisson Regression for Binary Outcomes (52 Patients). SPSS for Starters and 2nd Levelers [Internet]. 2016 [cited 2024 Aug 6];273–7. Available from: https://link.springer.com/chapter/10.1007/978-3-319-20600-4_47

32. Tetanus | Encyclopedia.com [Internet]. [cited 2024 Jul 29]. Available from: https://www.encyclopedia.com/medicine/diseases-and-conditions/pathology/tetanus#3435100772
33. Fauveau V, Mamdani M, Steinglass R, Koblinsky M. Maternal tetanus: Magnitude, epidemiology and potential control measures. International Journal of Gynecology & Obstetrics. 1993 Jan;40(1):3–12.
34. Health Organization W. WHO vaccine-preventable diseases: monitoring system 2009 global summary. 2009 [cited 2024 Jun 22]; Available from: www.who.int/vaccines-documents/
35. Rahman M, Chen LC, Chakraborty J, Yunus M, Chowdhury AI, Sarder AM, et al. Use of tetanus toxoid for the prevention of neonatal tetanus. 1. Reduction of neonatal mortality by immunization of non-pregnant and pregnant women in rural Bangladesh. Bull World Health Organ. 1982;60(2):261–7.
36. Amin MB, Roy N, Meem AE, Hossain E, Aktarujjaman M. Trends and determinants of taking tetanus toxoid vaccine among women during last pregnancy in Bangladesh: Country representative survey from 2006 to 2019. PLoS One. 2022;17(10):e0276417.
37. Roisin AJ, Prazuck T, Tall F, Sanou J, Cot M, Ballereau F V. Risk factor for neonatal tetanus in west Burkina Faso: a case control study. Eur J Epidemiol [Internet]. 1996 Oct;12(5):535–7. Available from: http://www.ncbi.nlm.nih.gov/pubmed/8905317
38. Gitta SN, Wabwire-Mangen F, Kitimbo D, Pariyo G, Centers for Disease Control and Prevention (CDC). Risk factors for neonatal tetanus--Busoga region, Uganda, 2002-2003. MMWR Suppl. 2006 Apr 28;55(1):25–30.
39. Chai F, Prevots DR, Wang X, Birmingham M, Zhang R. Neonatal tetanus incidence in China, 1996-2001, and risk factors for neonatal tetanus, Guangxi Province, China. Int J Epidemiol. 2004 Jun;33(3):551–7.
40. Singh A, Pallikadavath S, Ogollah R, Stones W. Maternal tetanus toxoid vaccination and neonatal mortality in rural north India. PLoS One. 2012;7(11):e48891.
41. Ronsmans C, Chowdhury ME, Alam N, Koblinsky M, El Arifeen S. Trends in stillbirths, early and late neonatal mortality in rural Bangladesh: the role of public health interventions. Paediatr Perinat Epidemiol. 2008 May;22(3):269–79.
42. Althabe F, Sosa C, Belizán JM, Gibbons L, Jacquerioz F, Bergel E. Cesarean section rates and maternal and neonatal mortality in low-, medium-, and high-income countries: an ecological study. Birth. 2006 Dec;33(4):270–7.
43. Mohamed SOO, Ahmed EM. Prevalence and determinants of antenatal tetanus vaccination in Sudan: a cross-sectional analysis of the Multiple Indicator Cluster Survey. Trop Med Health. 2022 Jan 10;50(1):7.
44. Rutstein SO. Effects of preceding birth intervals on neonatal, infant and under-five years mortality and nutritional status in developing countries: evidence from the demographic and health surveys. Int J Gynaecol Obstet. 2005 Apr;89 Suppl 1:S7-24.
45. Arokiasamy P, Gautam A. Neonatal mortality in the empowered action group states of India: trends and determinants. J Biosoc Sci. 2008 Mar;40(2):183–201.
46. Reid A. Neonatal mortality and stillbirths in early twentieth century Derbyshire, England. Popul Stud (NY). 2001 Nov;55(3):213–32.
47. Bloom SS, Lippeveld T, Wypij D. Does antenatal care make a difference to safe delivery? A study in urban Uttar Pradesh, India. Health Policy Plan. 1999 Mar;14(1):38–48.

48. Chandrashekar S, Rao RS, Nair NS, Kutty PR. Socio-demographic determinants of antenatal care. Trop Doct. 1998 Oct;28(4):206–9.
49. Ram F, Singh A. Is antenatal care effective in improving maternal health in rural uttar pradesh? Evidence from a district level household survey. J Biosoc Sci. 2006 Jul;38(4):433–48.
50. Chaudhary S, Contag S. The effect of maternal age on fetal and neonatal mortality. Journal of Perinatology 2017 37:7 [Internet]. 2017 Mar 30 [cited 2024 Aug 7];37(7):800–4. Available from: https://www.nature.com/articles/jp201736
51. Fonseca SC, Flores PVG, Camargo KR, Pinheiro RS, Coeli CM. Maternal education and age: inequalities in neonatal death. Rev Saude Publica [Internet]. 2017 [cited 2024 Aug 7];51. Available from: https://pubmed.ncbi.nlm.nih.gov/29166446/
52. Chowdhury AMR, Bhuiya A, Chowdhury ME, Rasheed S, Hussain Z, Chen LC. The Bangladesh paradox: exceptional health achievement despite economic poverty. Lancet. 2013 Nov 23;382(9906):1734–45.
53. Yaya S, Kota K, Buh A, Bishwajit G. Antenatal visits are positively associated with uptake of tetanus toxoid and intermittent preventive treatment in pregnancy in Ivory Coast. BMC Public Health. 2019 Dec 6;19(1):1467.
54. Yaya S, Kota K, Buh A, Bishwajit G. Prevalence and predictors of taking tetanus toxoid vaccine in pregnancy: a cross-sectional study of 8,722 women in Sierra Leone. BMC Public Health. 2020 Jun 5;20(1):855.

Table -1: The row-wise proportional distribution across various categories

	Neonatal mortality status		Total n (%)	P-value
	Dead n (%)	Alive n (%)		
**TT vaccinated**				
No	202(3.3)	5934(96.7)	6136(54.1)	0.255
Yes	152(2.9)	5055(97.1)	5207(45.9)	
**TT doses taken**				
None	202(3.3)	5934(96.7)	6136(54.1)	0.254
1 injection	84(3.2)	2532(96.8)	2616(23.1)	
2 injections or more (adequate doses)	68(2.6)	2519(97.4)	2587(22.8)	
**Cesarean section**				
Yes	117(3.1)	3687(96.9)	3804(67.2)	<0.001
No	90(4.8)	1769(95.2)	1859(32.8)	
**Gender**				
Boy	330(2.7)	11776(97.3)	12106(51.7)	0.028
Girl	257(2.3)	11039(97.7)	11296(48.3)	
**Antenatal care (ANC) visits**				
Less than 4	154(3.0)	5053(97.0)	5207(57.1)	0.077
Above 4	110(3.0)	3591(97.0)	3701(40.6)	
Don’t know	12(5.7)	200(94.3)	212(2.3)	
**Division**				
Barisal	49(2.4)	2020(97.6)	2069(8.8)	0.004

Chittagong	119(2.4)	4778(97.6)	4897(20.9)	
Dhaka	91(2.0)	4508(98.0)	4599(19.7)	
Khulna	70(2.2)	3158(97.8)	3228(13.8)	
Mymensingh	37(2.7)	1341(97.3)	1378(5.9)	
Rajshahi	71(2.9)	2379(97.1)	2450(10.5)	
Rangpur	76(2.7)	2689(97.3)	2765(11.8)	
Sylhet	74(3.7)	1942(96.3)	2016(8.6)	
**Women education level**				
Primary or secondary	527(2.7)	19236(97.3)	19763(84.5)	0.001
Higher secondary	60(1.6)	3579(98.4)	3639(15.6)	
**Birth order**				
1st parity	240(2.8)	8248(97.2)	8488(36.3)	
2nd parity	264(2.2)	11944(97.8)	12208(52.2)	0.002
3rd parity or more	72(3.1)	2459(96.9)	2531(10.8)	
**Women age**				
15-19	50(2.9)	1678(97.1)	1728(7.4)	
20-24	202(2.9)	6868(97.1)	7070(30.2)	0.055
25-29	170(2.5)	6711(97.5)	6881(29.4)	
30-34	91(1.9)	4707(98.1)	4798(20.5)	
35-39	55(2.5)	2188(97.5)	2243(9.6)	
40-44	16(2.9)	535(97.1)	551(2.4)	
45-49	3(2.3)	128(97.7)	131(0.6)	

**Wealth index**				
Poorest	306(2.9)	10355(97.1)	10661(45.6)	<0.001
Middle	214(2.4)	8613(97.6)	8827(37.7)	
Richest	67(1.7)	3847(98.3)	3914(16.7)	
~~Total~~				

Table 2. Factor Associated with Neonatal Mortality

Characteristics	**Crude IRR (95% CI)**	**p-value**	**Adjusted IRR (95% CI)**	**p-value**
**TT vaccinated**				
Yes	1		1	
No	1.19(0.92,1.52)	0.181	1.36(0.96,1.93)	0.081
**Cesarean section**				
Yes	1		1	
No	1.48(1.04,2.10)	0.028	1.32(0.93,1.88)	0.123
**Gender**				
Boy	1.23(1.02,1.47)	0.030	1.27(0.92,1.75)	0.151
Girl	1		1	
**ANC visits**				
Less than 4	1		1	
Above 4	1.10(0.80,1.50)	0.556	1.20(0.79,1.83)	0.395
Don't know	1.22(0.60,2.46)	0.578	1.51(0.68,3.39)	0.314
**Division**				
Barisal	0.81(0.54,1.22)	0.309	1.18(0.57,2.44)	0.659
Chittagong	0.88(0.63,1.22)	0.428	1.13(0.58,2.20)	0.729
Dhaka	0.76(0.54,1.08)	0.123	1.31(0.70,2.45)	0.392
Khulna	0.86(0.58,1.27)	0.446	1.05(0.54,2.05)	0.884
Mymensingh	0.90(0.59,1.37)	0.612	1.28(0.51,3.24)	0.599
Rajshahi	1.03(0.72,1.48)	0.854	1.25(0.63,2.49)	0.526
Sylhet	1.45(0.97,2.16)	0.071	1.66(0.65,4.25)	0.289
Rangpur	1		1	
**Women education level**				
Primary or secondary	1.73(1.27,2.34)	<0.001	1.86(1.16,2.99)	0.011
Higher secondary	1		1	
**Birth order**				

1st parity	1.29(1.07,1.56)	0.007	1.11(0.75,1.64)	0.611
2nd parity	1		1	
3rd parity	1.42(1.08,1.88)	0.014	1.96(0.95,4.03)	0.067
**Women Age**				
15-19	1.61(1.09,2.37)	0.017	1.36(0.60,3.09)	0.461
20-24	1.68(1.25,2.27)	0.001	1.89(0.93,3.86)	0.081
25-29	1.50(1.11,2.02)	0.009	1.62(0.86,3.04)	0.134
30-34	1		1	
35-39	1.34(0.90,1.99)	0.143	1.09(0.46,2.54)	0.851
40-44	1.76(0.95,3.25)	0.073	2.35(0.44,12.45)	0.317
45-49	1.95(0.63,6.05)	0.247	4.05(0.97,16.95)	0.055
**Wealth index**				
Poorest	1.70(1.25,2.33)	0.001	1.77(0.93,3.38)	0.085
Middle	1.39(1.01,1.90)	0.041	1.58(0.98,2.54)	0.060
Richest	1		1	

* **IRR = Incidence Rate Ratio**

Supplementary materials

Table 3: Analysis results for examining against Tetanus doses taken by mothers

Characteristics	Crude IRR (95% CI)	p-value	Adjusted IRR (95% CI)	p-value
**TT doses taken**				
None	1		1	
1 injection	0.84(0.64,1.09)	0.197	0.77(0.54,1.11)	0.160
2 injections or more (adequate doses)	0.87(0.57,1.32)	0.498	0.54(0.29,1.01)	0.054
**Cesarean section**				
Yes	1		1	
No	1.48(1.04,2.10)	0.030	1.32(0.93,1.88)	0.122
**Gender**				
Boy	1.3(1.02,1.47)	0.030	1.26(0.92,1.75)	0.155
Girl	1		1	
**ANC visits**				
Less than 4	1		1	
Above 4	1.01(0.80,1.51)	0.560	1.20(0.77,1.83)	0.401
Don’t know	1.22(0.61,2.46)	0.580	1.51(0.67,3.38)	0.320
**Division**				
Barisal	0.81(0.54,1.22)	0.309	1.18(0.57,2.44)	0.663
Chittagong	0.88(0.63,1.22)	0.428	1.14(0.58,2.22)	0.709
Dhaka	0.76(0.54,1.08)	0.123	1.32(0.71,2.46)	0.388
Khulna	0.86(0.58,1.27)	0.446	1.05(0.54,2.04)	0.898
Mymensingh	0.90(0.59,1.37)	0.612	1.28(0.51,3.24)	0.603

Rajshahi	1.03(0.72,1.48)	0.854	1.24(0.62,2.47)	0.547
Sylhet	1.45(0.97,2.16)	0.071	1.66(0.65,4.25)	0.287
Rangpur	1		1	
**Women education level**				
Primary or secondary	1.73(1.27,2.34)	<0.001	1.86(1.15,2.98)	0.011
Higher secondary	1		1	
**Birth order**				
1st parity	1.29(1.07,1.55)	<0.001	1.10(0.75,1.64)	0.617
2nd parity	1		1	
3rd parity	1.42(1.08,1.88)	0.010	1.96(0.95,4.02)	0.068
**Women Age**				
15-19	1.61(1.09,2.37)	0.010	1.36(0.60,3.09)	0.458
20-24	1.68(1.25,2.27)	<0.001	1.90(0.93,3.87)	0.078
25-29	1.50(1.11,2.02)	0.010	1.62(0.86,3.04)	0.135
30-34	1		1	
35-39	1.34(0.90,1.99)	0.140	1.08(0.46,2.52)	0.855
40-44	1.76(0.95,3.25)	0.070	2.37(0.45,12.56)	0.311
45-49	1.95(0.63,6.05)	0.250	4.60(0.99,21.29)	0.051
**Wealth index**				
Poorest	1.71(1.25,2.33)	0.010	1.78(0.94,3.40)	0.079
Middle	1.39(1.01,1.90)	0.040	1.59(0.99,2.56)	0.054
Richest	1		1	

*** IRR = Incidence Rate Ratio**

Date: 14 September 2024

To:
Dr Md. Moyazzem Hossain
Academic Editor
PLOS ONE

Subject: Submission of Revised Manuscript

Dear Dr Hossain,
Thank you for your invitation to submit a revised version of our manuscript "Maternal Tetanus Toxoid Immunization and Neonatal Mortality in the Context of Bangladesh: a study of association between neonatal mortality and tetanus toxoid vaccination" (PONE-D-24-08202R1).
According to the comments made by the respected reviewers we modified our manuscript. Below we address all comments made by the reviewers' point-by-point (reviewers' comments in italic, our responses in non-italic plain font and page numbers are according to the track change version of the revised manuscript). We look forward to your positive response.

Sincerely,

On behalf of all authors,
Dr. Md Jamal Uddin (Corresponding authors)
Professor of Biostatistics and Epidemiology,
Department of Statistics,
Shahjalal University of Science and Technology,
Sylhet, Bangladesh.
Phone: +8801716972846
Email: jamal-sta@sust.edu

Comments from reviewer:

Reviewer #1

Once again, I say well done to the authors for the job done. I still have my bias about what the manuscript is trying to prove. My reason for this bias is based on the outcome of the statistical analysis of this study which showed some factors as significant contributors to neonatal mortality. However, the authors focus on one factor that depends on those significant factors and far more important causes of neonatal mortality. Also, the authors need to go over the manuscript as the manuscript still has some grammatical errors.

Response: We appreciate the reviewer's attention to this matter. Certainly, we concur with the reviewer's assessment of this specific incident, which indicates that a number of substantial factors contribute to neonatal mortality. It makes sense that the factors would have an effect on each other since this is a multi-variable model. This means that these factors not only have an effect on neonatal mortality but also on the maternal tetanus vaccine. Although several significant factors have an impact on each other and on outcome variable, our primary objective is to demonstrate the relationship between maternal tetanus vaccination and neonatal mortality. Consequently, we solely concentrated on demonstrating the influence of maternal tetanus vaccination on neonatal mortality even though, it is possible to argue that neonatal mortality is also influenced by all of these other significant factors. We also discussed these factors in the results section of the manuscript.

Furthermore, we implemented extensive revisions targeting grammatical problems and punctuation, and we even altered sentence structure in certain places. Such as some punctuation

and grammatical errors in Abstract at page no. 1 (line no. 19 to 20, line no. 22 to 25), page no. 1 and 2 (line no. 27 to 33), in Introductory section at page no. 3 (line no. 56, 58), and page no. 4 (line no. 63, 76, and 77), page no. 5 (line no. 86 to 88, 105), Materials and Method section at page no. 8 (line no. 157), page no. 9 (line no. 184, 189), in Result section at page no. 15 (line no. 295, 299 to 300, 302 to 304, 306, 314 to 316), page no. 16 (line 318, 320, 324, 326, 328, 330 to 342), in Discussion section at page no. 17 and 18 (line no. 380, 382 to 385, 388 to 390, 394), page no. 19 (line no. 409 to 413), in Limitation section at page no. 21 (line no. 446, 450), in Conclusion section at page no. 32 (line no. 461 to 464, 466 to 468, 471).

Some sentences were also replaced with different sentence structures, like in the Abstract section at page no. 2 (line 25 to 33), "The adjusted incidence rate ratio (IRR) was 1.36, which indicates that there was a 36% higher risk of neonatal mortality for those children whose mothers did not take TT (IRR = 1.36, p = 0.081). We also found that women from middle-class households (IRR = 1.58, 95% CI = 0.98, 2.54) and women with higher parity (IRR = 1.96, 95% CI = 0.95, 4.03) also had a higher risk of newborn fatalities. A comparable trend has been observed regarding the correlation between the number of tetanus doses administered and neonatal mortality, where it also emphasizes the importance of receiving adequate doses (a minimum of 2 doses of tetanus vaccine) to mitigate neonatal mortality (adjusted IRR = 0.54, 95% CI = 0.29, 1.01) in comparison to no doses received." Additionally, in line no. 34, 35 we added, "Administering a minimum of one maternal tetanus dose significantly lowers the risk of neonatal mortality."

In Introductory section at page 3 (line no. 40 to 54), "Tetanus is an acute infectious disease that continues to pose a significant public health challenge. It occurs when the umbilical stump is covered with contaminated material or when non-sterile tools are used to cut the umbilical cord. It

can complicate surgery, intramuscular injections, gangrene, burns, ulcers, necrotic snakebites, middle ear infections, septic abortions, and childbirth. Tetanus can impact individuals across all age groups; however, it presents a particularly significant risk to newborns and pregnant women lacking adequate tetanus-toxoid vaccinations. In such cases, the disease can often result in death or significant health complications.". At page 3 (line no. 59 to 60), "However, it should be noted that tetanus cannot be completely eliminated due to the presence of spores in the natural environment.". At page no.4 (Line no. 61 to 75), "Maternal and neonatal tetanus (MNT) is a significant public health issue that impacts mothers and their newborns. It is prevalent when women give birth in unsanitary conditions and do not receive the complete series of tetanus toxoid (TT) vaccinations. So, an effective approach to reducing the spread of the disease is through the immunization of newborns and the women who are planning to become pregnant. This immunization can be achieved by vaccination with tetanus-toxoid-containing vaccines (TTCV). Maternal and neonatal tetanus (MNT) protection through vaccination ensures that a baby remains protected from the disease during the initial two months of life.". At page no.6 (line no. 109 to 114), "Tetanus is still prevalent and a major cause of death in low-income countries, despite considerable World Health Organization programs that effectively target maternal and newborn disease. As opposed to low-income countries (LICs), High-Income Countries (HICs) like the United States of America, England, and Denmark have long since managed tetanus-related newborn mortality. A few Low and Middle-Income Countries (LMICs), like Bangladesh, Afghanistan, and Nepal, are also making progress in lowering neonatal tetanus fatality rates even though the pace of reduction is very low.". And (line no. 115 to 29), "Effective intervention programs have been implemented in these countries, which include improved cord care, increased tetanus toxoid coverage for pregnant women, adoption of safe birth practices, and implementation

of postpartum care procedures. Despite some successes in countries such as Bangladesh and Nepal, low- and middle-income countries (LMICs) still face challenges in reducing infant mortality caused by neonatal tetanus. According to our knowledge, no study ever before was able to give a clear picture of the influence of maternal tetanus toxoid vaccination in reducing neonatal mortality in Bangladesh. The objective of our study was to investigate the potential link between neonatal mortality and tetanus toxoid immunization among women aged 15-49 in Bangladesh. This study will assist policymakers and planners in making more informed and effective decisions regarding this important issue.”.

In Materials and Method section at page no. 7 (line no. 135 to 172), “The research was conducted using a cross-sectional study design, utilizing the accessible data from the Bangladesh Multiple Indicator Cluster Survey (MICS) conducted by the Bangladesh Bureau of Statistics (BBS) in collaboration with the United Nations International Children's Emergency Fund (UNICEF) in 2019. The survey was carried out from January 19 to June 1, 2019. The survey sample was selected through a two-stage, stratified cluster sampling method in order to recruit the participants. The 2011 Bangladesh Census of Population and Housing was used as the sampling frame for this study. The enumeration areas (EAs) selected for the census enumeration were the primary sampling units (PSUs) selected during the initial stage. In each sample, a household listing was completed as part of the research process. In the second stage, a sample of households was selected for further analysis. The sample was created to generate estimates for various variables concerning women and children across the entire country, including both urban and rural areas in all 64 districts of the seven administrative divisions. The survey consisted of a sample of 64,400 households and approximately 3,220 primary sampling units (PSUs). The households were thoroughly interviewed by trained interviewers using questionnaires that covered reproductive

history, prenatal and postnatal care, family planning, and demographic characteristics. The datasets were made available to the public for access and were specifically collected from sampling units (PSUs).

Children who have passed away during the neonatal period or within the first month after birth may be considered as eligible cases for participation in the study. Moreover, it is important to note that the study subjects were specifically chosen to include children who had a high risk of newborn mortality, even though they were still alive at the time of the survey. Births that occurred prior to 2014 or five years before the survey are not classified as cases.". At page no. 9 (line no. 174 to 186), "The outcome variable is the child's survival status in the neonatal stage, which is binary and classified as either 0 (for alive) or 1 (for death). The analysis utilized data on the age at which mortality events occurred among live births that took place in the five-year period prior to the MICS 2019 survey. The variable indicating whether or not a neonatal death occurred was recoded using the standard procedures outlined in the SPSS syntax files provided by UNICEF. These recoded data were used to create the outcome variable for the research analysis.". At page no. 10(line no. 202,203), "Poisson regression performs better because of its log transformation, which improves results stability and interpretability.".

In Ethics statement at page no. 10 and 11 (line no. 2016 to 223), "The analysis utilized publicly accessible datasets from health surveys, ensuring that all identifiable personal information was removed. The analysis conducted in this study utilized secondary data sources, thereby exempting it from the need for ethical review approval from the relevant institutions. Nonetheless, the methods employed in MICS underwent a thorough review and received approval from both UNICEF and BBS.".

In Result section at page no. 12, 13 (line no. 227 to 270), "The most recent MICS (2019) survey data were integrated into this study. Among the 23,402 neonatal births, there were 587 neonatal fatalities, accounting for approximately 2.54% of the total neonatal births (Fig. 1). Approximately 45.9% of women received at least one dose of tetanus toxoid immunization during their most recent pregnancy, while 22.8% of women received the adequate number of doses. A multitude of factors played a role in the occurrence of early newborn deaths. In the univariate analysis presented in Table 1, it was observed that 67.2% of women underwent cesarean section, while the percentage of normal deliveries stood at 32.8%. Notably, the death rate associated with cesarean sections was low, recorded at 1.7%. Observations indicate that neonatal mortality rates are elevated in boys at 2.7%, compared to 2.3% in girls during delivery. It is worth mentioning that the neonatal mortality rates were 1.7% for mothers who had fewer than four antenatal care (ANC) visits. On the other hand, mothers who had four or more ANC visits had a slightly lower neonatal mortality rate of 1.21%. Furthermore, a minor percentage of mothers (0.13%) reported uncertainty regarding the number of antenatal care visits they had attended. Notably, a substantial correlation in univariate analysis has been identified between division and neonatal mortality, with mothers from the Sylhet division experiencing the highest neonatal mortality rate at 3.7%. It is important to highlight that education significantly influences neonatal mortality rates. Women with primary or secondary education experienced a 2.7% rate of neonate deaths, while those with higher secondary education had a rate of 1.6%. Birth order appears to be an important factor, as second-parity births are linked to increased neonatal mortality, highlighting the need for further investigation into the underlying causes. Women with increased birth parity experience elevated neonatal mortality, which constitutes another critical factor. The wealth index of the respondents indicates that 45.6% came from poor families, 37.2%

were from the middle class, and 16.7% belonged to the rich category. The neonatal mortality rates are observed to be 2.9% for the poorest, 2.4% for the middle class, and 1.7% for the richest segments of the population. Finally, it is noteworthy that a considerable proportion of women aged 20–24 experienced a significant rate of newborn infant mortality, recorded at 2.9%.". A sentence has been moved from 256 to 241, "A similar trend has been found in the relationship between the number of tetanus doses taken and neonatal mortality that has been given in Appendix 1, which also describes taking adequate doses (at least 2 doses of tetanus vaccine) to reduce neonatal mortality (Crude IRR = 0.87, 95%CI = 0.57, 1.32) compared to none taken host." And also (line no. 274 to 290), "Table 1 displays the mortality status row proportions across different covariate categories. It is evident from the table that the respondents classified in the lowest wealth index category experience the highest percentage of neonatal deaths at 2.9%, in contrast to the richest group at 1.7% and the middle class at 2.4%. The Sylhet division exhibits the highest proportion of neonatal mortality at 3.7%, closely followed by the Rajshahi division at 2.9%. The mortality rate is notably elevated in individuals with a third parity or higher, recorded at 3.1%. Additionally, mothers who experienced natural births exhibit a greater percentage of neonatal deaths (4.8%) in contrast to those who underwent cesarean sections (3.1%). Finally, the mothers who received the tetanus toxoid vaccine exhibited a lower neonatal mortality rate of 3.3% compared to the 2.9% observed in non-vaccinated mothers during their last pregnancy.".

In Discussion section at page no. 17, 18 (line no. 347 to 370), "Tetanus demonstrates a significant threat to human life (32). Despite significant advancements over the past twenty years in reducing global tetanus-related fatalities, neonatal tetanus has garnered considerable focus, while maternal tetanus remains largely underexplored (15,33). This survey aims to identify the impact of neonatal mortality in relation to tetanus toxoid vaccination among pregnant women

aged 15 to 49 years. The findings indicate that maternal vaccination plays a significant role in decreasing neonatal death rates.

Previous investigations have not explored the relationship between neonatal mortality and tetanus toxoid immunization in conjunction with other essential covariates. Our primary results demonstrate that women who obtain at least one dose of the tetanus vaccine substantially reduce the likelihood of neonatal death in their children. Furthermore, around 2.54% of neonatal deaths in Bangladesh are associated with pregnant women who did not receive at least one dose of the tetanus toxoid vaccination. In contrast, about 45.9% of women have received at least one dose of the vaccine, which could serve as valuable data for initiatives aimed at enhancing vaccination rates.".

In the conclusion, "Furthermore, Women with higher education levels, those in the wealthiest quintile, and those with greater parity were all significantly linked to reduced odds of newborn mortality." was added in the line 367.
