# Supplementary material for: A study of association between maternal tetanus toxoid immunization and neonatal mortality in the context of Bangladesh
Source: PLoS One. 2025 Jan 17;20(1):e0316939. doi: 10.1371/journal.pone.0316939 (PMC11741588; doi:10.1371/journal.pone.0316939)
Supplement: Attachment — Submitted filename: Response to reviewers.docx [file pone.0316939.s003.docx]

Date: 14 July 2024

To:

Dr Md. Moyazzem Hossain

Academic Editor

PLOS ONE

**Subject: Submission of Revised Manuscript**

Dear Dr Hossain,

Thank you for your invitation to submit a revised version of our manuscript "Maternal Tetanus Toxoid Immunization and Neonatal Mortality in the Context of Bangladesh: a study of association between neonatal mortality and tetanus toxoid vaccination" (PONE-D-24-08202).

According to the comments made by the respected reviewers we modified our manuscript. Below we address all comments made by the reviewers’ point-by-point (reviewers’ comments in italic, our responses in non-italic plain font and page numbers are according to the track change version of the revised manuscript). We look forward to your positive response.

Sincerely,

On behalf of all authors,

Dr. Md Jamal Uddin (Corresponding authors)

Professor of Biostatistics and Epidemiology,

Department of Statistics,

Shahjalal University of Science and Technology,

Sylhet, Bangladesh.

Phone: +8801716972846

Email: jamal-sta@sust.edu

**Comments from Editor:**

1. The title may be “A Study of Association Between Neonatal Mortality and Maternal Tetanus Toxoid Immunization in the Context of Bangladesh”.

**Response: We thank the editor for their appreciation of our manuscript and for providing valuable comments. We replace the title with “A Study of Association Between Neonatal Mortality and Maternal Tetanus Toxoid Immunization in the Context of Bangladesh”.**

2. In Methods of Abstract, the authors are requested to mention the sample size.

**Response: Thanks to the editor for highlighting this issue. It has been solved by adding “**The dataset consists of 23,402 cases, among them 587 cases resulted in the infant death.**” in methods section of the abstract (Page 1, Line no. 19).**

3. It is necessary to update the Introduction section based on the statistics and studies focusing on this topic in the context of Bangladesh.

**Response: We can't thank you enough for this valuable insight. A paragraph (page 4 to 5, line no. 74 to 85) has been added in the introduction section on the fifth para.**

**“**The Bangladesh Demographic and Health Survey reported neonatal mortality rates of 28 per 1,000 live births in 2014 and 30 in 2017, while under-five mortality rates were 46 per 1,000 in 2014 and 45 in 2017. The current rate is significantly higher than the global average of 6.6 per 1,000 live births in 2015 and exceeds the 7.4 per 1,000 averages in developing countries and neighboring countries like India and Nepal. The WHO reports that preterm birth causes 30% of global neonatal deaths, sepsis or pneumonia 27%, birth asphyxia 23%, congenital abnormalities 6%, neonatal tetanus 4%, diarrhea 3%, and other causes 7%. An exploratory study in rural Bangladesh found that tetanus accounts for 42% of newborn deaths. According to another study, out of 330 infant deaths, 112 of them fit the tetanus case description. There were 24.4 neonatal deaths for every 1000 live births in Bangladesh, based on 6748 neonatal fatalities spread throughout four districts (Thakurgaon, Jamalpur, Moulvibazar, and Narail), with a combined population of 6.7 million. Despite TT2's estimated 45% efficacy, maternal history of receiving two doses of the tetanus toxoid did not lower the risk in Bangladesh.**”**

4. The authors write “Factors significant in bivariate models (p < 0.2) were included in the multivariable model…” Justify the use of p<0.2 and add references.

**Response: Thank you for your time. We fixed this noted problem at page 7, line no. 138 to 140 with a reference.**

5. Add a strengths and limitations section.

**Response: Thank you the editor for indicating this oversight. A strength and limitation section has been added to page 16 to 17 (Line 3018 to 346).**

**“****Limitations and Strengths**

**Strengths**

To our knowledge, this study is the first in Bangladesh to investigate the relationship between maternal tetanus toxoid vaccination and neonatal mortality. We employed appropriate data analysis techniques, taking into consideration all intricate survey designs. Findings from this work can be used to inform future research, policy, and clinical practice and to benchmark progress. One of the benefits of this study is the sizeable and nationally representative sample size, and the results are applicable to the entire country. The information was gathered using the most recent survey. Our results would surely pique interest in additional research and educate decision-makers about the gaps in tetanus care that need to be filled.

**Limitations**

However, despite the several strengths, the existence of bias resulting from different survey time points and the cross-sectional character of the data cannot be confirmed. Some variables that were insignificant (at 90% C.I.) but still important for the research study have been considered in the model. It is crucial to keep in mind that the mortality that is being discussed here includes deaths from all causes. Regretfully, our analysis lacks particular data that isolates the risk exclusively for newborn mortality. Notwithstanding this restriction, the trend that has been seen points to a higher risk of death for those who have not had the TT vaccination, however statistical significance is not attained. This realization emphasizes how crucial it is to conduct additional studies to determine the specific effect of the TT vaccine on mortality outcomes, especially neonatal death. To offer more precise information, future research focusing on infant mortality rates and any confounding factors is required.

The level of significance was deemed to be fairly high. In addition, the study's drawback also emerges from the fact that we had little control over the correlated variables to include in the analysis due to the secondary data source we employed. Conclusions regarding a causal association, the relative contributions of immunization prior versus during the most recent pregnancy, or the best possible ways to increase coverage are not possible due to the cross-sectional character of this investigation. In addition, the dependent variable for newborn death has a large number of missing values, making it impossible to include them in the analysis. This could lead to bias, and the variable varies over time, potentially changing the claimed association in longitudinal studies.”

**Thank you again for pointing this out.**

**We have also added some information into result section (page 11, line 220 to 225) as**

**“**Crude incidence rate ratios (IRRs) for death are shown in the characteristics table for women who had tetanus toxoid (TT) immunization vs those who did not. When compared to the vaccinated group, the unvaccinated group's crude IRR for death was 1.185 at first, with a p-value of 0.18, indicating no discernible difference in mortality. However, after adjusting the other factors in the model, the crude IRR rose to 1.364 with a p-value of 0.08. Although this is not statistically significant at the traditional cutoff point of p < 0.05, this adjusted IRR suggests an elevated risk of MTT on infant mortality. **”**

REVIEWER #1:

*I salute the authors for the great efforts put into the work. The manuscript however has lots of errors of syntax and semantics.*

*I will advice the authors to take a very good look at the findings of the study and possibly modify the title of the work as it is very difficult to link maternal tetanus vaccination with all-cause neonatal mortality as they have tried to do in this study.*

*Maternal tetanus vaccination does not have effect on several other causes of neonatal mortality, so it will be difficult to attribute maternal tetanus vaccination alone to neonatal mortality.*

**Response: We are delighted by the reviewer’s feedback and thank you.**

**Syntax and semantics related errors has been diminished.**

**Title has been replaced with the one that Editor advised.**

**We managed to give a clear picture related to other causes of neonatal mortality on the Strength and limitation section, page 16 (Line 329 to 338).**

*Some of the references were not properly cited, they should be corrected.*

**Response: Thank you very much for the concern. We are pleased to inform you that it has been corrected.**

REVIEWER #2:

*1. This is not a study finding, rather this is the findings from the existing public domain health survey datasets. I think it will be more appropriate if the manuscript title also reflect this. Authors need to change the title as “Maternal Tetanus Toxoid Immunization and Neonatal Mortality in Bangladesh: The findings from a retrospective analysis of the data from Bangladesh Multiple Indicator Cluster Survey”*

**Response: We are honored for your comments. We have already changed the title to “A Study of Association Between Neonatal Mortality and Maternal Tetanus Toxoid Immunization in the Context of Bangladesh”. It was suggested by the editor.**

*2. Authors write this as study in the abstract (line 35, 49) and in many place in the manuscript. Please change the study as analysis or survey.*

**Response: Thank you. We have changed the study as analysis or survey where necessary.**

*3. Referencing need massive correction. In discussion 32 ref came earlier than 29, 31…*

**Response: Thank you very much. Reference section has been renovated by re-arranging with proper care.**

*4. Define ‘adequate doses’ in the method section not in result section (line 168)*

**Response: Thank you again. It has been removed from the results section and added in method section page 6 to 7, line 129 and 130.**

*5. Please reproduce the figure 1 with addition of alive and death group for all divisions within the vaccinated and non-vaccinated participants and calculate p value here.*

**Response: Thank you. Figure 1 i.e, “Tetanus toxoid vaccination status across different divisions” is reproduced now depicting alive and death groups for all divisions within the vaccinated and non-vaccinated participants and also indicating the p-values. It is thus renamed to Figure 2, as we have introduced another chart in the manuscript named Figure 1, depicting the “Proportion of dead and alive neonates”.**

*6. Please remove figure 3. This figure has no relation with TT vaccine.*

**Response: Thank You. Figure 3 has been removed.**

*7. Massive English correction is needed for the full manuscript.*

**Response: Thank you. We have revised the manuscript and fixed the grammatical errors.**

*8. There is no limitation explain in the paper. Like, this is an analysis from the existing public domain health survey datasets. Prospective study may reveal more in depth information.*

**Response: Thank you for the insight, we have added a strength and limitations section in the manuscript.**
