# Supplementary material for: A study of association between maternal tetanus toxoid immunization and neonatal mortality in the context of Bangladesh
Source: PLoS One. 2025 Jan 17;20(1):e0316939. doi: 10.1371/journal.pone.0316939 (PMC11741588; doi:10.1371/journal.pone.0316939)
Supplement: Attachment — Submitted filename: Response to reviewers.docx [file pone.0316939.s004.docx]

Date: 14 September 2024

To:

Dr Md. Moyazzem Hossain

Academic Editor

PLOS ONE

**Subject: Submission of Revised Manuscript**

Dear Dr Hossain,

Thank you for your invitation to submit a revised version of our manuscript "Maternal Tetanus Toxoid Immunization and Neonatal Mortality in the Context of Bangladesh: a study of association between neonatal mortality and tetanus toxoid vaccination" (PONE-D-24-08202R1).

According to the comments made by the respected reviewers we modified our manuscript. Below we address all comments made by the reviewers’ point-by-point (reviewers’ comments in italic, our responses in non-italic plain font and page numbers are according to the track change version of the revised manuscript). We look forward to your positive response.

Sincerely,

On behalf of all authors,

Dr. Md Jamal Uddin (Corresponding authors)

Professor of Biostatistics and Epidemiology,

Department of Statistics,

Shahjalal University of Science and Technology,

Sylhet, Bangladesh.

Phone: +8801716972846

Email: jamal-sta@sust.edu

**Comments from reviewer:**

Reviewer #1

*Once again, I say well done to the authors for the job done. I still have my bias about what the manuscript is trying to prove. My reason for this bias is based on the outcome of the statistical analysis of this study which showed some factors as significant contributors to neonatal mortality. However, the authors focus on one factor that depends on those significant factors and far more important causes of neonatal mortality. Also, the authors need to go over the manuscript as the manuscript still has some grammatical errors.*

**Response:** We appreciate the reviewer's attention to this matter. Certainly, we concur with the reviewer's assessment of this specific incident, which indicates that a number of substantial factors contribute to neonatal mortality. It makes sense that the factors would have an effect on each other since this is a multi-variable model. This means that these factors not only have an effect on neonatal mortality but also on the maternal tetanus vaccine. Although several significant factors have an impact on each other and on outcome variable, our primary objective is to demonstrate the relationship between maternal tetanus vaccination and neonatal mortality. Consequently, we solely concentrated on demonstrating the influence of maternal tetanus vaccination on neonatal mortality even though, it is possible to argue that neonatal mortality is also influenced by all of these other significant factors. We also discussed these factors in the results section of the manuscript.

Furthermore, we implemented extensive revisions targeting grammatical problems and punctuation, and we even altered sentence structure in certain places. Such as some punctuation and grammatical errors in Abstract at page no. 1 (line no. 19 to 20, line no. 22 to 25), page no. 1 and 2 (line no. 27 to 33), in Introductory section at page no. 3 (line no. 56, 58), and page no. 4 (line no. 63, 76, and 77), page no. 5 (line no. 86 to 88, 105), Materials and Method section at page no. 8 (line no. 157), page no. 9 (line no. 184, 189), in Result section at page no. 15 (line no. 295, 299 to 300, 302 to 304, 306, 314 to 316), page no. 16 (line 318, 320, 324, 326, 328, 330 to 342), in Discussion section at page no. 17 and 18 (line no. 380, 382 to 385, 388 to 390, 394), page no. 19 (line no. 409 to 413), in Limitation section at page no. 21 (line no. 446, 450), in Conclusion section at page no. 32 (line no. 461 to 464, 466 to 468, 471).

Some sentences were also replaced with different sentence structures, like in the Abstract section at page no. 2 (line 25 to 33), “The adjusted incidence rate ratio (IRR) was 1.36, which indicates that there was a 36% higher risk of neonatal mortality for those children whose mothers did not take TT (IRR = 1.36, p = 0.081). We also found that women from middle-class households (IRR = 1.58, 95% CI = 0.98, 2.54) and women with higher parity (IRR = 1.96, 95% CI = 0.95, 4.03) also had a higher risk of newborn fatalities. A comparable trend has been observed regarding the correlation between the number of tetanus doses administered and neonatal mortality, where it also emphasizes the importance of receiving adequate doses (a minimum of 2 doses of tetanus vaccine) to mitigate neonatal mortality (adjusted IRR = 0.54, 95% CI = 0.29, 1.01) in comparison to no doses received.” Additionally, in line no. 34, 35 we added, “Administering a minimum of one maternal tetanus dose significantly lowers the risk of neonatal mortality.”

In Introductory section at page 3 (line no. 40 to 54), “Tetanus is an acute infectious disease that continues to pose a significant public health challenge. It occurs when the umbilical stump is covered with contaminated material or when non-sterile tools are used to cut the umbilical cord. It can complicate surgery, intramuscular injections, gangrene, burns, ulcers, necrotic snakebites, middle ear infections, septic abortions, and childbirth. Tetanus can impact individuals across all age groups; however, it presents a particularly significant risk to newborns and pregnant women lacking adequate tetanus-toxoid vaccinations. In such cases, the disease can often result in death or significant health complications.”. At page 3 (line no. 59 to 60), “However, it should be noted that tetanus cannot be completely eliminated due to the presence of spores in the natural environment.”. At page no.4 (Line no. 61 to 75), “Maternal and neonatal tetanus (MNT) is a significant public health issue that impacts mothers and their newborns. It is prevalent when women give birth in unsanitary conditions and do not receive the complete series of tetanus toxoid (TT) vaccinations. So, an effective approach to reducing the spread of the disease is through the immunization of newborns and the women who are planning to become pregnant. This immunization can be achieved by vaccination with tetanus-toxoid-containing vaccines (TTCV). Maternal and neonatal tetanus (MNT) protection through vaccination ensures that a baby remains protected from the disease during the initial two months of life.”. At page no.6 (line no. 109 to 114), “Tetanus is still prevalent and a major cause of death in low-income countries, despite considerable World Health Organization programs that effectively target maternal and newborn disease. As opposed to low-income countries (LICs), High-Income Countries (HICs) like the United States of America, England, and Denmark have long since managed tetanus-related newborn mortality. A few Low and Middle-Income Countries (LMICs), like Bangladesh, Afghanistan, and Nepal, are also making progress in lowering neonatal tetanus fatality rates even though the pace of reduction is very low.”. And (line no. 115 to 29), “Effective intervention programs have been implemented in these countries, which include improved cord care, increased tetanus toxoid coverage for pregnant women, adoption of safe birth practices, and implementation of postpartum care procedures. Despite some successes in countries such as Bangladesh and Nepal, low- and middle-income countries (LMICs) still face challenges in reducing infant mortality caused by neonatal tetanus. According to our knowledge, no study ever before was able to give a clear picture of the influence of maternal tetanus toxoid vaccination in reducing neonatal mortality in Bangladesh. The objective of our study was to investigate the potential link between neonatal mortality and tetanus toxoid immunization among women aged 15-49 in Bangladesh. This study will assist policymakers and planners in making more informed and effective decisions regarding this important issue.”.

In Materials and Method section at page no. 7 (line no. 135 to 172), “The research was conducted using a cross-sectional study design, utilizing the accessible data from the Bangladesh Multiple Indicator Cluster Survey (MICS) conducted by the Bangladesh Bureau of Statistics (BBS) in collaboration with the United Nations International Children's Emergency Fund (UNICEF) in 2019. The survey was carried out from January 19 to June 1, 2019. The survey sample was selected through a two-stage, stratified cluster sampling method in order to recruit the participants. The 2011 Bangladesh Census of Population and Housing was used as the sampling frame for this study. The enumeration areas (EAs) selected for the census enumeration were the primary sampling units (PSUs) selected during the initial stage. In each sample, a household listing was completed as part of the research process. In the second stage, a sample of households was selected for further analysis. The sample was created to generate estimates for various variables concerning women and children across the entire country, including both urban and rural areas in all 64 districts of the seven administrative divisions. The survey consisted of a sample of 64,400 households and approximately 3,220 primary sampling units (PSUs). The households were thoroughly interviewed by trained interviewers using questionnaires that covered reproductive history, prenatal and postnatal care, family planning, and demographic characteristics. The datasets were made available to the public for access and were specifically collected from sampling units (PSUs).

Children who have passed away during the neonatal period or within the first month after birth may be considered as eligible cases for participation in the study. Moreover, it is important to note that the study subjects were specifically chosen to include children who had a high risk of newborn mortality, even though they were still alive at the time of the survey. Births that occurred prior to 2014 or five years before the survey are not classified as cases.”. At page no. 9 (line no. 174 to 186), “The outcome variable is the child's survival status in the neonatal stage, which is binary and classified as either 0 (for alive) or 1 (for death). The analysis utilized data on the age at which mortality events occurred among live births that took place in the five-year period prior to the MICS 2019 survey. The variable indicating whether or not a neonatal death occurred was recoded using the standard procedures outlined in the SPSS syntax files provided by UNICEF. These recoded data were used to create the outcome variable for the research analysis.”. At page no. 10(line no. 202,203), “Poisson regression performs better because of its log transformation, which improves results stability and interpretability.”.

In Ethics statement at page no. 10 and 11 (line no. 2016 to 223), “The analysis utilized publicly accessible datasets from health surveys, ensuring that all identifiable personal information was removed. The analysis conducted in this study utilized secondary data sources, thereby exempting it from the need for ethical review approval from the relevant institutions. Nonetheless, the methods employed in MICS underwent a thorough review and received approval from both UNICEF and BBS.”.

In Result section at page no. 12, 13 (line no. 227 to 270), “The most recent MICS (2019) survey data were integrated into this study. Among the 23,402 neonatal births, there were 587 neonatal fatalities, accounting for approximately 2.54% of the total neonatal births (Fig. 1). Approximately 45.9% of women received at least one dose of tetanus toxoid immunization during their most recent pregnancy, while 22.8% of women received the adequate number of doses. A multitude of factors played a role in the occurrence of early newborn deaths. In the univariate analysis presented in Table 1, it was observed that 67.2% of women underwent cesarean section, while the percentage of normal deliveries stood at 32.8%. Notably, the death rate associated with cesarean sections was low, recorded at 1.7%. Observations indicate that neonatal mortality rates are elevated in boys at 2.7%, compared to 2.3% in girls during delivery. It is worth mentioning that the neonatal mortality rates were 1.7% for mothers who had fewer than four antenatal care (ANC) visits. On the other hand, mothers who had four or more ANC visits had a slightly lower neonatal mortality rate of 1.21%. Furthermore, a minor percentage of mothers (0.13%) reported uncertainty regarding the number of antenatal care visits they had attended. Notably, a substantial correlation in univariate analysis has been identified between division and neonatal mortality, with mothers from the Sylhet division experiencing the highest neonatal mortality rate at 3.7%. It is important to highlight that education significantly influences neonatal mortality rates. Women with primary or secondary education experienced a 2.7% rate of neonate deaths, while those with higher secondary education had a rate of 1.6%. Birth order appears to be an important factor, as second-parity births are linked to increased neonatal mortality, highlighting the need for further investigation into the underlying causes. Women with increased birth parity experience elevated neonatal mortality, which constitutes another critical factor. The wealth index of the respondents indicates that 45.6% came from poor families, 37.2% were from the middle class, and 16.7% belonged to the rich category. The neonatal mortality rates are observed to be 2.9% for the poorest, 2.4% for the middle class, and 1.7% for the richest segments of the population. Finally, it is noteworthy that a considerable proportion of women aged 20–24 experienced a significant rate of newborn infant mortality, recorded at 2.9%.”. A sentence has been moved from 256 to 241, “A similar trend has been found in the relationship between the number of tetanus doses taken and neonatal mortality that has been given in Appendix 1, which also describes taking adequate doses (at least 2 doses of tetanus vaccine) to reduce neonatal mortality (Crude IRR = 0.87, 95%CI = 0.57, 1.32) compared to none taken host.” And also (line no. 274 to 290), “Table 1 displays the mortality status row proportions across different covariate categories. It is evident from the table that the respondents classified in the lowest wealth index category experience the highest percentage of neonatal deaths at 2.9%, in contrast to the richest group at 1.7% and the middle class at 2.4%. The Sylhet division exhibits the highest proportion of neonatal mortality at 3.7%, closely followed by the Rajshahi division at 2.9%. The mortality rate is notably elevated in individuals with a third parity or higher, recorded at 3.1%. Additionally, mothers who experienced natural births exhibit a greater percentage of neonatal deaths (4.8%) in contrast to those who underwent cesarean sections (3.1%). Finally, the mothers who received the tetanus toxoid vaccine exhibited a lower neonatal mortality rate of 3.3% compared to the 2.9% observed in non-vaccinated mothers during their last pregnancy.”.

In Discussion section at page no. 17, 18 (line no. 347 to 370), “Tetanus demonstrates a significant threat to human life (32). Despite significant advancements over the past twenty years in reducing global tetanus-related fatalities, neonatal tetanus has garnered considerable focus, while maternal tetanus remains largely underexplored (15,33). This survey aims to identify the impact of neonatal mortality in relation to tetanus toxoid vaccination among pregnant women aged 15 to 49 years. The findings indicate that maternal vaccination plays a significant role in decreasing neonatal death rates.

Previous investigations have not explored the relationship between neonatal mortality and tetanus toxoid immunization in conjunction with other essential covariates. Our primary results demonstrate that women who obtain at least one dose of the tetanus vaccine substantially reduce the likelihood of neonatal death in their children. Furthermore, around 2.54% of neonatal deaths in Bangladesh are associated with pregnant women who did not receive at least one dose of the tetanus toxoid vaccination. In contrast, about 45.9% of women have received at least one dose of the vaccine, which could serve as valuable data for initiatives aimed at enhancing vaccination rates.”.

In the conclusion, “Furthermore, Women with higher education levels, those in the wealthiest quintile, and those with greater parity were all significantly linked to reduced odds of newborn mortality.” was added in the line 367.
