# Supplementary material for: A study of association between maternal tetanus toxoid immunization and neonatal mortality in the context of Bangladesh
Source: PLoS One. 2025 Jan 17;20(1):e0316939. doi: 10.1371/journal.pone.0316939 (PMC11741588; doi:10.1371/journal.pone.0316939)
Supplement: Attachment — Submitted filename: Response to reviewers.docx [file pone.0316939.s006.docx]

Date: 11 November 2024

To:

Dr Md. Moyazzem Hossain

Academic Editor

PLOS ONE

**Subject: Submission of Revised Manuscript**

Dear Dr Hossain,

Thank you for your invitation to submit a revised version of our manuscript "Maternal Tetanus Toxoid Immunization and Neonatal Mortality in the Context of Bangladesh: a study of association between neonatal mortality and tetanus toxoid vaccination" (PONE-D-24-08202R2).

According to the comments made by the respected reviewers we modified our manuscript. Below we address all comments made by the reviewers’ point-by-point (reviewers’ comments in italic, our responses in non-italic plain font and page numbers are according to the track change version of the revised manuscript). We look forward to your positive response.

Sincerely,

On behalf of all authors,

Dr. Md Jamal Uddin (Corresponding authors)

Professor of Biostatistics and Epidemiology,

Department of Statistics,

Shahjalal University of Science and Technology,

Sylhet, Bangladesh.

Phone: +8801716972846

Email: jamal-sta@sust.edu

**Comments from reviewer:**

**Reviewer #1**

Reviewer: I believe that the authors can arrange the age in ascending order (Page 4, line no. 69).

Response: Thank you for notifying us point by point. We have re-arrange the age in ascending order.

Reviewer: This should be 15 - 19 in order to avoid overlap with the next age range 20 – 24 (page 7, line 149).

Response: We thank the editor for pointing out this one. The correction on this has been done.

Reviewer: party (page 10, line no. 208.)

Response: Thanks. This spelling mistake has been taken care of.

Reviewer: I believe that the authors inadvertently substituted the values written in this sentence. The authors need to review this sentence please (Page 10, line no. 210-212).

Response: Again, we thank the editor whole heartedly for pointing this major mistake. The values were interchanged and written as, “Finally, the mothers who received the tetanus toxoid vaccine exhibited a lower neonatal mortality rate of 2.9% compared to the 3.3% observed in non-vaccinated mothers during their last pregnancy.”

Reviewer: The bracket should be removed (page 11, line 224).

Response: Thank you. All the brackets from all figure number has been removed in line 217, 221, 224.

Reviewer: This should be corrected - compared to taking none (page 12, line no. 249).

Response: Thanks. This has been corrected.

Reviewer: Is this the true finding of this study? The authors need to review this sentence as it contradicts the next referenced sentence (page 14, line no. 310 - 311).

Response: Thank you again. This issue has been taken care of and corrected as, “Our study revealed that women with higher parity have higher odds of neonatal mortality compared to those with lower parity. According to our findings, women with three or more parity had 43% grater odds comparing to women with second parity. This is supported by similar findings from previous studies that have found higher parity to be a significant predictor of neonatal mortality (44–46).”

Reviewer: quantile or quintile? (page 15, line 327)

Response: Thank you. “quantile” was replaced with “quintile”.

Reviewer: This sentence needs to be rephrased please. (page 15, line no 330-331)

Response: We can’t thank you enough. The line “A policy and programming requirement to ensure that all pregnant women receive at least one dose of prenatal medication” is rephrased as “The implementation of policies and programs that guarantee all pregnant women receive a minimum of one dosage of prenatal medication” at line no. 331.
